# Immunological responses and gut microbial shifts in *Phthorimaea absoluta* exposed to *Metarhizium anisopliae* isolates under different temperature regimes

**DOI:** 10.3389/fmicb.2023.1258662

**Published:** 2023-11-09

**Authors:** Felix Muendo Maingi, Komivi Senyo Akutse, Inusa Jacob Ajene, Kevin Mbogo Omolo, Fathiya Mbarak Khamis

**Affiliations:** ^1^International Centre of Insect Physiology and Ecology (icipe), Nairobi, Kenya; ^2^Department of Biochemistry, Jomo Kenyatta University of Agriculture and Technology, Nairobi, Kenya; ^3^Unit for Environment Sciences and Management, North-West University, Potchefstroom, South Africa

**Keywords:** tomato leaf miner, *Metarhizium anisopliae*, hemocytes, microbiome, temperature regimes

## Abstract

The invasive tomato leaf miner, *Phthorimaea absoluta,* is conventionally controlled through chemical insecticides. However, the rise of insecticide resistance has necessitated sustainable and eco-friendly alternatives. Entomopathogenic fungi (EPF) have shown potential due to their ability to overcome resistance and have minimal impact on non-target organisms. Despite this potential, the precise physiological mechanisms by which EPF acts on insect pests remain poorly understood. To attain a comprehensive understanding of the complex physiological processes that drive the successful control of *P. absoluta* adults through EPF, we investigated the impacts of different *Metarhizium anisopliae* isolates (ICIPE 665, ICIPE 20, ICIPE 18) on the pest’s *survival*, cellular immune responses, and gut microbiota under varying temperatures. The study unveiled that ICIPE 18 caused the highest mortality rate among *P. absoluta* moths, while ICIPE 20 exhibited the highest significant reduction in total hemocyte counts after 10 days at 25°C. Moreover, both isolates elicited notable shifts in *P. absoluta’s* gut microbiota. Our findings revealed that ICIPE 18 and ICIPE 20 compromised the pest’s defense and physiological functions, demonstrating their potential as biocontrol agents against *P. absoluta* in tomato production systems.

## 1. Introduction

*Phthorimaea absoluta* (Lepidoptera: Gelechiidae) is an invasive insect pest of tomato and other solanaceous plants, which is rapidly expanding its geographic range (Mutamiswa et al., 2017). The pest is reported to have originated in the central highlands of Peru in South America in 1917 (Shiberu and Getu, 2018; Chang and Metz, 2021). In Africa, the *P. absoluta* invasion was first reported in Morocco, Tunisia, and Algeria in 2008, and later in 2014 in Witu and Mpeketoni fields in Lamu County, Kenya (Tonnang et al., 2015; Kinyanjui et al., 2021). Since then, the pest has spread to all other major tomato-growing regions in Kenya (Kinyanjui et al., 2021). *P. absoluta* poses a significant threat to the sustainable production of tomatoes, resulting in yield losses ranging from 80 to 100% with substantial price increases for tomatoes in the market (Chepchirchir et al., 2021; Kinyanjui et al., 2021). The high yield and economic losses are linked to *P. absoluta’*s high reproduction potential, short life cycle, the nocturnal activity of adults, and the larval stage’s concealed feeding behavior inside the leaf mesophyll, making it the most devastating insect pest present in tomato-producing areas in sub-Saharan Africa (Tonnang et al., 2015; Birhan, 2018).

Chemical pesticides are the most commonly used management practices against *P. absoluta* because they are easily accessible, cheap, and quick-acting (Skinner et al., 2014). However, farmers often mis- and overuse these pesticides as they are applied prophylactically without following the labels and even without detecting the pest (Chidege et al., 2016; Pandey et al., 2023). Heavy application of pesticides has negative consequences on the environment, leads to the development of insecticide resistance, and endangers the production of healthy foods due to the accumulation of chemical residues (Rwomushana et al., 2019). Due to these shortcomings, alternative and environmentally friendly control approaches against *P. absoluta* are being developed to supplement or entirely replace chemical pesticides (Agbessenou et al., 2020).

The search for alternatives to chemical pesticides has turned to natural microbial pathogens of insects (Skinner et al., 2014). Naturally-occurring entomopathogens have been shown to regulate insect populations in the wild (Ding et al., 2020). Several entomopathogenic fungi (EPF) species, such as *M. anisopliae* and *Beauveria bassiana*, have been adopted as biological control agents against insect pests (Qu and Wang, 2018; Akutse et al., 2020a,b). Due to their negligible effect on non-target organisms and easy mass production, EPF are considered the next-generation control agent for insect pests (Jiang et al., 2020). *M. anisopliae* isolates (ICIPE 665, ICIPE 18, ICIPE 20) (Agbessenou et al., 2021) have recently been identified as potential control agents for the sustainable management of *P. absoluta* adults (Akutse et al., 2020a,b). However, their efficacy against insect pests is affected by abiotic factors such as temperature, humidity, light, and pH (Dimbi et al., 2009; Silva et al., 2020). The relatively slow mode of action of EPF compared to chemical insecticides has sometimes hindered their adoption and widespread application (Wei et al., 2017). In addition, insect pests have evolved defense mechanisms to counter the infection (Qu and Wang, 2018). Understanding the physiological mechanisms by which EPF exerts control over *P. absoluta* can lead to more targeted and effective pest control strategies (Wang et al., 2021).

The EPF infection starts with the attachment of spores to the outer layer of the insect’s cuticle. The spores then germinate, penetrate the cuticle, and grow inside the insect’s hemocoel (Wang et al., 2021). Upon infection, the insect senses the invading pathogen and activates cellular and humoral immune responses (Gwokyalya and Altuntaş, 2019). Cellular immune responses are activated immediately after infection, while humoral responses appear hours post-infection (Zhong et al., 2017). Cellular immunity is mediated by hemocytes, which directly recognize invading pathogens or indirectly recognize humoral receptors that bind to and opsonize the invader’s surface. Hemocytes protect the host from invading pathogens through various immune responses, including melanization, nodulation, encapsulation, phagocytosis, and release of antimicrobial peptides (Eleftherianos et al., 2021). However, EPF releases chemical metabolites such as destruxins and beauvericin that paralyze the immunity of the host insects (Jiang et al., 2020; Kryukov et al., 2021). This circular battle results in an evolutionary arms race between insect hosts and pathogens (Qu and Wang, 2018). In addition, the insect gut contains a diverse community of commensal bacteria, the microbiome, which influence the host immune system by modulating immune cell development, maintaining gut barrier integrity, and secreting metabolites that affect immune responses (Liu et al., 2013; Gichuhi et al., 2020; Zeng et al., 2022). These interactions promote tolerance beneficial to microbes while guarding against pathogens, impacting overall immune function and disease susceptibility (Liu et al., 2013; Zeng et al., 2022). The gut microbiome is involved in digestion, detoxification of allelochemicals, and stress responses (Kryukov et al., 2021). It also protects the insect from pathogen infection by inhibiting fungal growth and differentiation through systemic effects such as producing metabolites or signaling molecules that enter the hemocoel, activating immune responses and creating an environment less conducive to fungal growth (Wei et al., 2017; Zhang et al., 2017). Most studies investigating host-pathogen models have focused on the evolutionary and functional aspects, leaving a knowledge gap about the physiological mechanisms underpinning the efficacy of EPF as a control agent against insect pests (Gwokyalya et al., 2022). Thus, a significant lack of detailed information exists regarding the intricate physiological processes that drive the successful control of this pest through EPF. Understanding the physiological mechanisms by which EPF exerts control over *P. absoluta* can lead to more targeted and effective control strategies (Qu and Wang, 2018). This knowledge could help develop methods that optimize EPF applications, increasing their impact on pest populations while minimizing environmental impact (Yaneth et al., 2020).

To better understand these physiological mechanisms, we assessed the survival of *P. absoluta* adults upon exposure to *M. anisopliae* isolates (ICIPE 665, ICIPE 20, ICIPE18) previously identified for sustainable management of *P. absoluta* adults under different temperature conditions (15, 20, 25, and 30°C). Since the efficacy of EPF is affected by abiotic factors such as temperature (Onsongo et al., 2019), the experiment was conducted under varying temperature regimes to mimic the ecological conditions in which the insect is found in the field. Subsequently, we evaluated the cellular immune responses of surviving *P. absoluta* moths by focusing on cell population dynamics and morphological characterization of different hemocytes produced when adult tomato leafminers are exposed to the *M. anisopliae* isolates at varying temperature regimes. EPF triggers varying immune responses in insect pests (Zafar et al., 2020). Counting and identifying different hemocyte populations could offer insights into the types of immune cells activated and their abundance, shedding light on the specificity and intensity of the immune response (Marringa et al., 2014). Finally, we examined the effect of the most potent *M. anisopliae* isolates (ICIPE 20 and ICIPE 18) on the composition and diversity of bacterial communities within *P. absoluta* after infection at different temperature regimes.

The survival of *P. absoluta* following infection with *M. anisopliae* isolates is likely influenced by hemocyte counts and gut microbial diversity. Hemocyte counts reflect immune responses affecting the ability to combat the infection. On the other hand, gut microbial diversity could impact immune function, potentially influencing survival outcomes by affecting immune activation and overall physiological resilience. The relationship among these factors informs how immune reactions and microbial interactions contribute to survival outcomes in these host-pathogen interactions. Therefore, this study aimed to offer valuable insights into the impact of the *M. anisopliae* isolates on the biology of *P. absoluta* adults across various temperature conditions. This research will enhance our comprehension of the specific physiological mechanisms that underlie the effectiveness of EPF as potential control agents against *P. absoluta* adults, a crucial knowledge for developing targeted and sustainable pest control strategies.

## 2. Materials and methods

### 2.1. Phthorimaea absoluta

*Phthorimaea absoluta* colony was originally established from wild larvae and adults collected from infested tomatoes from Mwea (0°36′31.3″S 037°22′29.7″E) Kirinyaga County, Kenya, in June 2019. Emerged *P. absoluta* adults were reared on tomato plants in ventilated, sleeved Perspex cages (50 × 50 × 45 cm) before use in the experiments. The colony was rejuvenated every 3 months with infested tomato leaves collected from the field to avoid inbreeding. The adult *P. absoluta* were fed on a natural honey diet smeared on the top side of each cage. The colony was maintained under laboratory rearing conditions of 26 ± 2°C, 60% relative humidity, and 12:12 L.D photoperiod.

### 2.2. Fungal isolates

In this study, three isolates of *M. anisopliae* (ICIPE 665, ICIPE 20, and ICIPE 18) were used., The isolates were obtained from the germplasm of the Arthropod Pathology Unit (APU) at the International Centre of Insect Physiology and Ecology (ICIPE), where entomopathogenic fungi are kept. The isolates had been previously screened against *P. absoluta* (Akutse et al., 2020a,b). The strains were cultured on Sabouraud dextrose agar (SDA) from OXOID CM0041, made by Oxoid Ltd. in Basingstoke, UK, and kept in complete darkness at 25 ± 2°C. A sterile spatula was used to scrape the surface of 2–3-week-old cultures that had sporulated to obtain conidia. The conidia collected were suspended in 10 mL of sterile distilled water that contained 0.05% (w/v) Triton X-100 from MERCK KGaA in Darmstadt, Germany. The suspension was vortexed for 5 min to ensure that the conidia were evenly distributed and that any clumps of conidia were broken apart. Conidial counts and concentrations were determined using an improved Neubauer hemocytometer under a light microscope (Horner et al., 1995). Before assessing the viability, the concentration of conidia in the suspension was adjusted to 3 × 10^6^ conidia per milliliter using serial dilution.

To determine spore viability prior to conducting bioassays, 100 μL of 3 × 10^6^ conidia per milliliter suspension was spread evenly onto Petri dishes with a diameter of 9 cm containing SDA. A sterile microscope coverslip measuring 2 × 2 cm was then placed on top of the agar in each plate. The plates were sealed with parafilm membrane and subsequently incubated at 25 ± 2°C in complete darkness for observation after 16–20 h. To determine the percentage of conidia that had germinated, 100 conidia were randomly selected from the surface area enclosed by each coverslip and examined under a light microscope at 400× magnification. The approach described by Goetiel and Inglis (1997) was used for this purpose. Conidia were classified as having germinated if the germ tube length was at least twice the diameter of the conidium. Twenty replicates were used for each fungal isolate.

### 2.3. Effect of *Metarhizium anisopliae* fungal isolates on the survival of *Phthorimaea absoluta* adults

During our trials, temperatures above 30°C were fatal to the insects, given that all insects, including control groups, died during the first 2 days after their introduction into the incubator. The bioassays were therefore conducted at 15, 20, 25, and 30°C, representing the temperature range in which *P. absoluta* thrives in the field (Agbessenou et al., 2021). A total of 25 *P. absoluta* adults that were 1–3 days old were exposed to dry conidia of *M. anisopliae* strains using velvet-coated plastic jars (measuring 150 mm × 80 mm) as contamination devices. This was done following the procedure described by Migiro et al. (2010). The devices were contaminated with 1 g (equivalent to 0.15 × 10^9^ conidia/g) of dry conidia for each fungal isolate. This dosage was chosen based on previous studies by Akutse et al. (2020a,b) and Agbessenou et al. (2021). The moths were introduced into the contamination device for 3 min to pick up the spores. Control (untreated) insects were introduced into EPF-free contamination devices. Following a 3-min exposure to the conidia, the insects were moved into clean Perspex cages measuring 15 × 15 × 15 cm. Honey was then placed on the top of every cage to act as a food source. Each experiment consisted of 25 fungus-challenged moths per replicate, with three replicates per isolate, totalling 1,200 insects (challenged and control insects). Survival of adult *P. absoluta* in all the treatments was recorded daily for 10 days under different temperatures.

#### 2.3.1. Hemolymph collection

*Phthorimaea absoluta* adults challenged with *M. anisopliae* isolates were collected from incubators set at different temperature regimes (15, 20, 25, and 30°C) for hemolymph collection. Five live moths were picked at different time intervals (days 0, 1, 5, and 10) post-infection using a manual aspirator and transferred into glass vials with a lid to prevent them from escaping. While inside the glass vials, the moths were immersed in an ice bucket for 10 min to anesthetize/immobilize them before extracting the hemolymph. The moths that had been immobilized were surface sterilized using 70% ethanol, after which they were washed with distilled water to eliminate any remaining fur and scales before transferring them onto a paper towel at room temperature for 10 min to drain excess moisture and allow stabilization of normal physiological functions before hemolymph extraction. While holding the insect gently under a stereomicroscope, the insect’s wings and legs were gently chopped off using a sterile scalpel, taking care not to distort the hemocoel before transferring the insect onto parafilm under a stereomicroscope with the dorsal side facing up. A dissecting needle was placed lightly and held in place, and the posterior cuticle of the insect was opened using fine-pointed forceps as described by Hiroyasu et al. (2018). The hemolymph from the dissected insect was allowed to flow onto the paraffin film for 5 min and collected using a microcapillary tube. The hemolymph was utilized for subsequent bioassays. One part (1 μL) was used for total hemocyte counts, and the other (1 μL) for differential hemocyte counts.

#### 2.3.2. Total and differential hemocyte counts

The total hemocyte counts (THCs) assays were performed according to the procedure described by Altunta et al. (2012) with slight modifications. As explained above, 1 μL of hemolymph was obtained from each adult *P. absoluta*. The collected hemolymph was immediately introduced into a 1.5 microcentrifuge tube containing 9 μL of ice-cold anticoagulant (0.186 M NaCl, 0.098 M NaOH, 0.017 M Na₂EDTA, and 0.041 M citric acid (with a pH of 4.5)). The contents were mixed thoroughly. A volume of 10 μL of the mixture was added to an improved Neubauer hemocytometer, and the hemocytes were then counted under a light microscope (Leica DM 2500 LED, Leica Microsystems Wetzlar Germany). The THCs were reported as the number of hemocytes × 10^5^ conidiospore/ml hemolymph. The hemolymph was collected separately from five moths in each fungus-challenged group, and the experiment was replicated three times, resulting in a total of 15 samples (*n* = 15). Uninfected moths were used as controls, with five individuals in each of the three replicates. To assess the phenoloxidase activity, 10 μL of hemolymph was diluted in 100 μL of PBS (phosphate buffered saline) sample, and then incubated with 2 mg/mL L dihydroxyphenylalanine (L-DOPA) for 3 h. The measures of Phenoloxidase (PO) activity were read for 30 min with one-minute intervals at 490 nM in an Epoch™ 2 microplate spectrophotometer.

#### 2.3.3. Differential hemocyte counts

The Differential Hemocyte Counts (DHC) experiments were performed using a modified procedure described by Altunta et al. (2012). The hemolymph collection was done as described in Section 2.3 and then transferred to pre-cleaned microscope slides. After spreading the hemolymph on the slide, it was left to dry at room temperature, and then the hemocytes were fixed using a mixture of methanol and glacial acetic acid in a 3:1 ratio. After fixation, the hemocytes were treated with a solution of 5% Giemsa/PBS (v/v) for 45 min to stain them and rinsed with distilled water for 2 min. The stained hemocytes were viewed using a light microscope (Leica DM 2500 LED, Leica Microsystems Wetzlar Germany) and classified according to their types as described by Gupta (1979). In this experiment, 100 cells from each slide were randomly selected and counted. The different types of hemocytes were identified and recorded as percentages. The hemolymph was collected separately from five moths, and the process was repeated three times, making a total of 15 samples. The Leica LASZ software was used to capture images of the hemocytes, and Adobe Illustrator CC 2020 (version 24.2) was used to adjust the resolution of the images.

### 2.4. Effect of *Metarhizium anisopliae* isolates on the gut microbiome of *Phthorimaea absoluta* adults

#### 2.4.1. Sample collection

To assess the effect of the *M. anisopliae* isolates on the gut microbiota of *P. absoluta* adults, freshly emerged moths (1–3 days old) were exposed to the most potent *M. anisopliae* isolates (ICIPE 18 and ICIPE 20) before incubating them at the following temperature regimes (15, 20, and 25°C) as described above. At 30°C, the *P. absoluta* moths did not survive to day 10, including those in the control group, and they were excluded from the experiment. The two fungal isolates were selected due to their high virulence and capacity to induce the highest decline of total hemocyte counts in adult *P. absoluta*, as observed in the preceding bioassays. Five *P. absoluta* moths were randomly collected on days one and ten post-infection using a manual aspirator. The collected insects were preserved in 96% ethanol and kept at-80°C awaiting DNA extraction.

#### 2.4.2. Genomic DNA extraction and 16 rRNA metabarcoding

The samples collected above were used for genomic DNA extraction using the Isolate II Genomic DNA kit (Bioline, London, United Kingdom). The quality and concentration of the DNA extracts were checked using a Nanodrop 2000/2000c Spectrophotometer (Thermo Fischer Scientific, Wilmington, United States). DNA extracts within the A_260 nm_/A_280 nm_ ratio of 1.8–2.0 were eluted to a final volume of 50 μL and used for 16S rRNA sequencing.

For the library preparation with the 16S barcoding kit SQK-16S024, pooled DNA was utilized for multiplexing. The preparation of the library involved a PCR step that was performed with the following components:10 ng μL^−1^ of DNA template, 10 ng μL^−1^ of each 16S barcode, 5 × *MyTaq* reaction buffer (5 mM dNTPs, enhancer, stabilizer, and 15 mM MgCl_2_), *MyTaq* DNA polymerase (Bioline, London, United Kingdom). The experiments were conducted using a Master Cycler Nexus gradient thermal cycler (Eppendorf, Germany), where the reactions were set up in a total reaction volume of 50 μL. The thermal cycling conditions were as follows: an initial denaturation step of 2 min at 95°C, followed by 35 cycles at 95°C for 30 s, annealing at 55°C for 40 s, extension at 72°C for 1 min, and a final extension at 72°C for 10 min. Following the manufacturer’s instructions, the amplicons were purified using a Bioline purification kit. The purified amplicons were then pooled together and loaded into the flow cells for sequencing.

#### 2.4.3. Sequencing

Sequencing of the full length ~ 1,480 bp bacterial 16S rRNA was done using FLO-MIN106 flow cell for 4 h on the MinION sequencer [Oxford Nanopore Technology (ONT)]. The MinKNOW software (v20.10.3, ONT) was used as the platform to run the sequencing runs on the MinION device to allow the acquisition of raw data. The Albacore tool (v2.3.4, ONT) performed the live base calling on the ONT cloud. The FASTQ reads obtained from the sequencing were analyzed in real-time using the cloud-based platform EPI2ME Agent (v3.5.4–8,056,458; Metrichor, Oxford, UK) using the “What’s in my Pot” (WIMP v3.2.1) workflow. This turnkey tool encompasses demultiplexing quality checks and performs primary and secondary analysis, enabling rapid identification and classification of bacterial taxa in real-time up to the species level through the NCBI reference database.

### 2.5. Correlation between survival rates and immune responses of *Phthorimaea absoluta* post-*Metarhizium anisopliae* exposure

To investigate the relationship between the survival rate, THCs, DHCs, and gut microbial abundance of *P. absoluta* when infected with *M. anisopliae* isolate ICIPE 20 at different temperature regimes, a Pearson’s correlation analysis was conducted, and correlograms were generated for each of the parameters.

### 2.6. Data analysis

All statistical analyses were performed in R software (R Foundation for Statistical Computing, Vienna, Australia). Kaplan Meier Survival analysis was used to determine if the exposure of *P. absoluta* adults to *M. anisopliae* isolates negatively affected the survival of the insects using R-packages “survminer” (Kassambara et al., 2022) (Version 0.4.6), “ggplot2” (Hadley, 2022), “car” (John et al., 2022), and “plyr” (Wickham H 2022). Survival curves were fitted with the number of days *P. absoluta* adults were alive as the treatment function of the tested *M. anisopliae* isolates. Data were right-censored at 10 days post-infection. The survival analysis was conducted by generating Cox proportional Hazard Ratios (HR) with detection days as the response using the *Coxph* function with the “survival” package (Wickham, 2022) (version 3.4). A mixed-effects Cox interaction model was created to test for significant differences in surviving adult *P. absoluta*.

The impact of *M. anisopliae* isolates on *P*. *absoluta* hemocytes was investigated at different temperature regimes. The THCs and DHCs dynamics were evaluated at days 0, 1, 5, and 10 post-exposures. To establish a comparison between the treatments, a Tukey’s honestly significant difference (Tukey’s HSD) test was performed using R packages “multcomp” (Bretz et al., 2022) and “emmeans” (Russell et al., 2022) to determine the difference between hemocyte counts of challenged and unchallenged insects at *p ≤* 0.05. THC data were analyzed using a Generalized Linear Model (GLM) with a negative binomial regression procedure to take into account overdispersion. Differential hemocyte counts (plasmatocytes, granulocytes, prohemocytes, spherulocytes, oenocytes, and adipohemocytes) of *P. absoluta* were analyzed using the Betareg package (Cribari-neto and Gruen, 2022) with beta regression, which takes into account the beta-binomial distribution error considering the treatments (ICIPE 18, ICIPE20, ICIPE 665 and Control at days 0, 1, 5, and 10) as factors. The models were evaluated for their significance using deviance analysis through Chi-square tests. Tukey’s multiple comparison tests were performed with a significance level of p ≤ 0.05 to compare the means.

Since EPI2ME does not provide diversity parameters, data generated from the software was transferred to R (version 4.1) for alpha and beta statistics analysis. To select the most abundant taxa in each sample, a minimum abundance cut-off of 0.1% was applied. Taxa with cumulative read counts below the 0.1% cut-off were combined into “Others.” Comparing the cumulative read counts at the genus level provided better visualization of the taxonomic patterns in the data than at the species level. Alpha diversity statistics, including the Shannon-Wiener index, richness index, abundance index, and evenness index, were used to assess the bacterial diversity in each sample under the three different temperature regimes (i.e., 15, 20, and 25°C). To evaluate the variety of bacterial genera among the samples at different temperatures, the Jaccard dissimilarity index (Tang et al., 2021) was used to calculate beta diversity. Finally, a principal coordinate analysis (PCoA) was used to compute the inter-sample microbiome relationship using the “vegan” (Simpson et al., 2022) package.

## 3. Results

### 3.1. Effect of *Metarhizium anisopliae* fungal isolates on the survival of *Phthorimaea absoluta* adults

**Figure 1:**
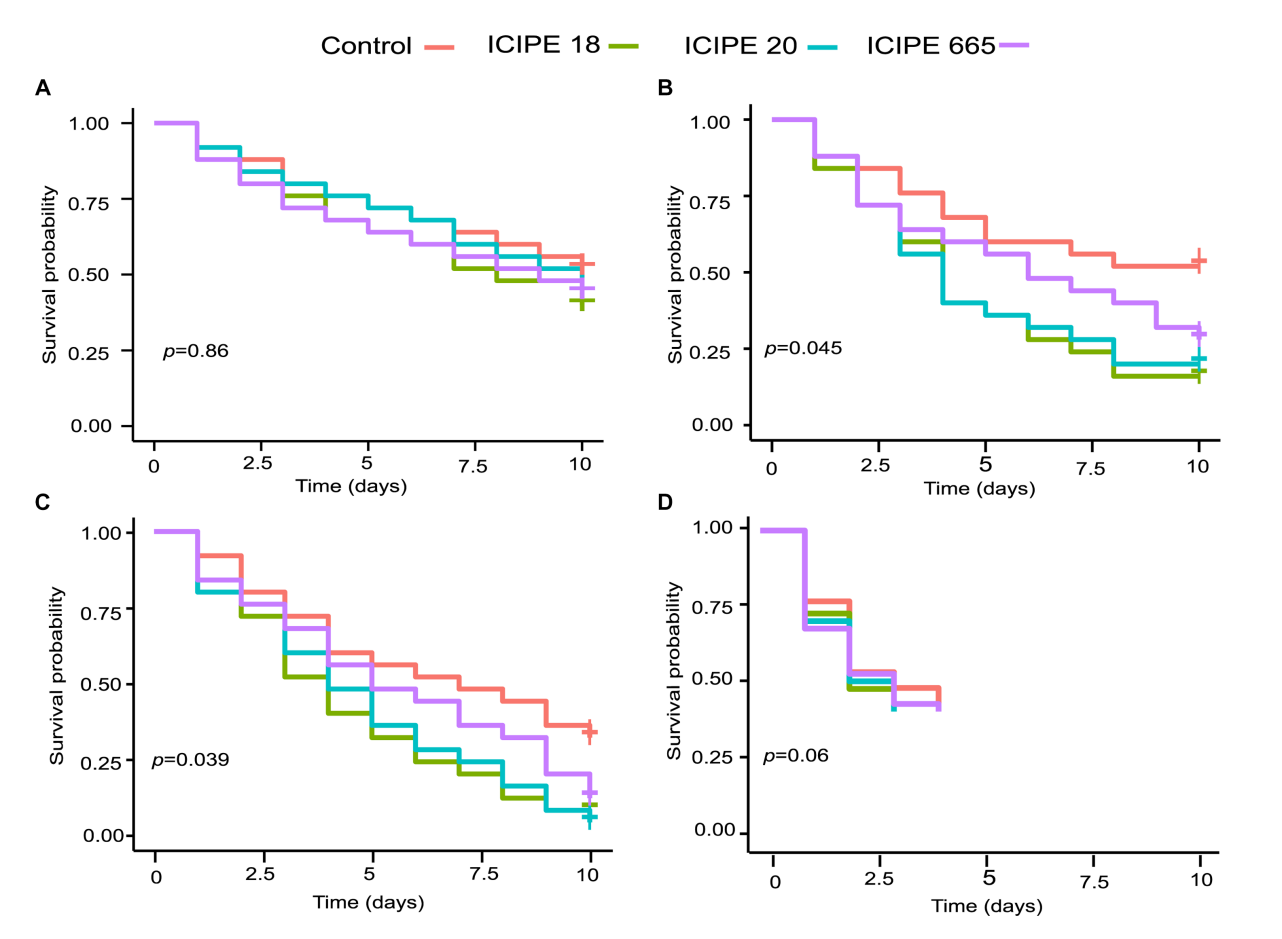
Kaplan-Meier survival curves for *Phthorimaea absoluta* adults exposed to *Metarhizium anisopliae* (ICIPE 18, ICIPE 20, ICIPE 665) at **(A)** 15°C, **(B)** 20°C, **(C)** 25°C and **(D)** 30°C. n=75 insects per treatment. ‘+’ indicates the right censorship.

The survival rate of fungus-challenged *P. absoluta* adults in bioassays conducted at 15°C was high, exceeding 75%. However, these survival rates did not show any statistically significant difference compared to the control groups (χ^2^ = 0.7, df = 3, *p =* 0.90; Figure 1A). Additionally, multiple comparisons of the hazard ratio values (HR) of the *M. anisopliae* isolates using Tukey contrasts did not show significant statistical difference when compared to the control groups (ICIPE 18 HR = 1.3791, *z* = 0.83, *p* = 0.41; ICIPE 20 HR = 1.1968, *z* = 0.46, *p* = 0.65; ICIPE 665 HR = 1.2834, *z* = 0.634, *p* = 0.53) (Supplementary Figure S1).

The survival rate of fungus-challenged *P. absoluta* moths in bioassays conducted at 20°C was statistically significant compared to the control group (χ^2^ = 8.1, df = 3, *p =* 0.04; Figure 1B). While it was observed that the three fungal isolates caused a decrease in the survival of *P. absoluta* moths compared to the control treatment (Tukey contrasts: *p* < 0.05), ICIPE 18 demonstrated the highest mortality surpassing that of ICIPE 20 and ICIPE 665 (HR = 2.5460, *z* = 2.566, *p* = 0.01; HR = 2.3615, *z* = 2.342, *p* = 0.02, HR = 1.7261, z = 1.464, *p* = 0.14 respectively) (Supplementary Figure S2).

Similarly, the survival rate of *P. absoluta* moths infected with *M. anisopliae* isolates in bioassays conducted at 25°C was remarkably low, less than 50%. Furthermore, statistical analysis (χ^2^ = 8.4, df = 3, *p* = 0.04; Figure 1C) indicated that this survival rate significantly differed from the control groups. Among the three isolates, ICIPE 18 caused the highest mortality rate, surpassing that induced by ICIPE 20 and ICIPE 665 (HR = 2.2817, *z* = 2.544, *p* = 0.011; HR = 2.1885, *z* = 2.439, *p =* 0.014; HR = 8.71, *z* = 3, *p =* 0.1654, respectively) (Supplementary Figure S3). However, in bioassays conducted at 30°C, the temperature was observed to be fatal to the insects, given that all insects in control died within the first 4 days after their introduction into the incubator (χ^2^ = 1.72, df = 3, *p* = 0.63) (Figure 1D).

### 3.2. Effect of *Metarhizium anisopliae* isolates on total hemocyte counts

**Figure 2:**
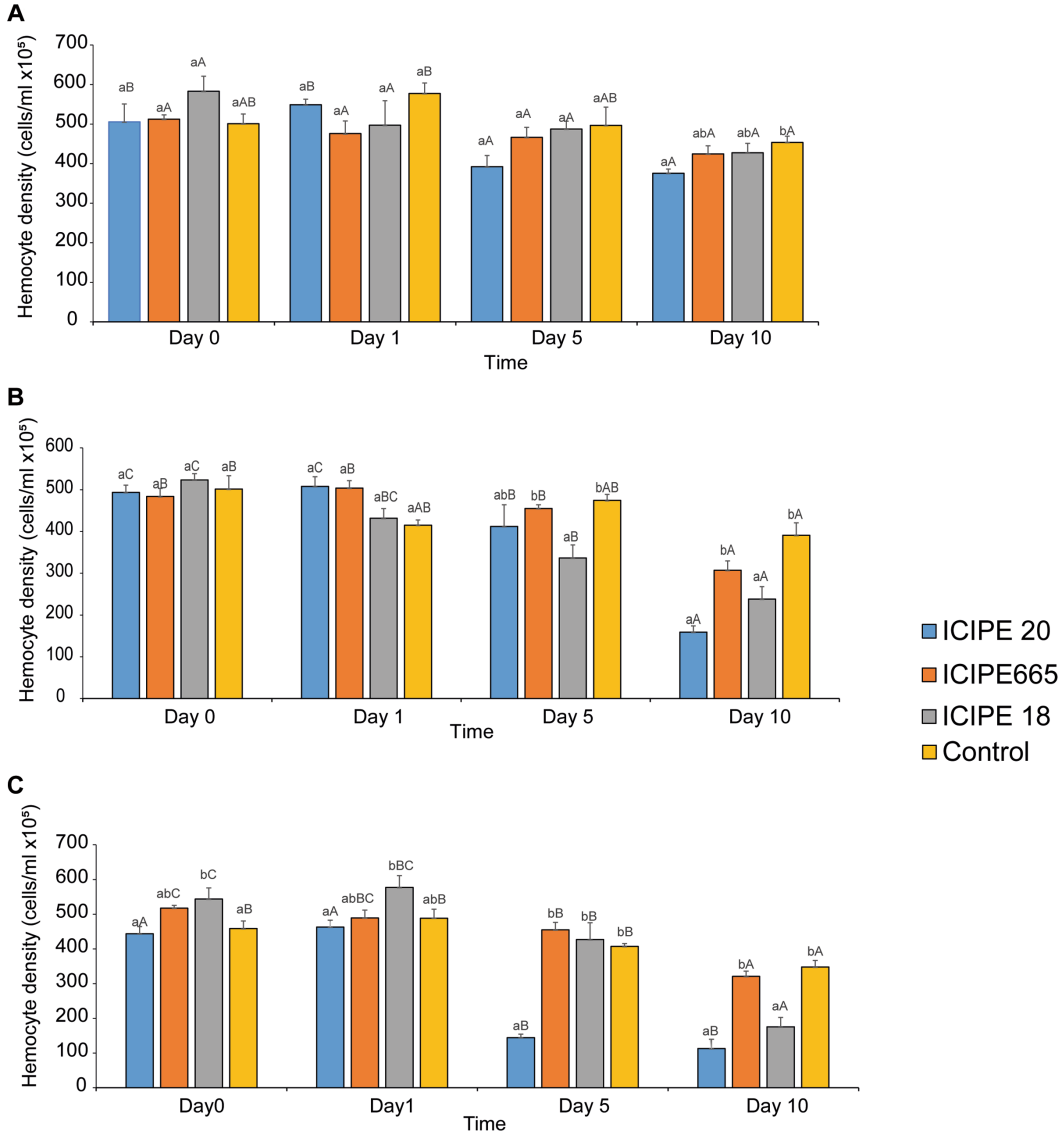
Mean host total hemocyte counts overtime in *Phthorimaea absoluta* in response to exposure to *Metarhizium anisopliae* at **(A)** 15°C, **(B)** 20°C, and **(C)** 25°C.

Analysis of total hemocyte counts (THCs) of fungus-challenged *P. absoluta* adults in bioassays conducted at 15°C did not exhibit any statistical significance on days 0, 1, and 5 (χ^2^ = 4.2255, df = 3, *p* = 0.2381; χ^2^ = 3.3706, df = 3, *p =* 0.3379; χ^2^ = 7.2447, df = 3, *p* = 0.0845; respectively) when compared to control groups. However, on the tenth-day post-infection, a notable disparity in the THCs was observed between *M. anisopliae*-infected *P. absoluta* adults and the control group counterparts. This difference was statistically significant, as indicated by test results (χ^2^ = 9.63, df = 3, *p* = 0.02) (Figure 2A; Supplementary Figure S4A). The most significant decline in THCs was observed in ICIPE 20-infected *P. absoluta* adults, followed by those challenged with ICIPE 665 and finally, those infected with ICIPE 18 (17.21, 6.4, 5.74%, respectively, relative to the control).

When THCs of fungus-challenged *P. absoluta* moths in bioassays conducted at 20°C were compared to the control groups, there was no statistically significant difference on days 0 and 1 post-infection (χ^2^ = 1.25 df = 3, *p =* 0.74; χ^2^ = 10.02, df = 3, *p =* 0.18, respectively). However, on both days 5 and 10 after infection, the THCs of the infected insects were significantly lower compared to the control group counterparts (χ^2^ = 19.44, df = 3, *p <* 0.01; χ^2^ = 42.78, df = 3, *p* < 0.001, respectively). Notably, on the fifth day, *P. absoluta* moths infected with ICIPE 18 exhibited the lowest THCs among the three isolates. They were followed by moths infected with ICIPE 20 and those challenged with ICIPE 665 (29.08, 13.17, and 4.12%, decline proportions, respectively, relative to the control). The highest decline in THCs was recorded on the tenth day after *M. anisopliae* infection. Adult *P. absoluta* infected with ICIPE 20 exhibited the most significant decrease in THCs, followed by those infected with ICIPE 18 and ICIPE 665 (59.33, 39.13, 21.48%, respectively, relative to the control) (Figure 2B; Supplementary Figure S4B).

In bioassays conducted at 25°C, the THCs of fungus-challenged *P. absoluta* moths were significantly fewer than those of control groups. This statistical difference was observed on days 0, 1,5, and 10 after the infections were initiated (χ^2^ = 13.66, df = 3, *p <* 0.001; χ^2^ = 11.16, df = 3, *p =* 0.01; χ^2^ = 117.69, df = 3, *p <* 0.001; and χ^2^ = 47.30, df = 3, *p <* 0.001, respectively). Across the four time intervals, adult *P. absoluta* infected with ICIPE 20 exhibited lower THCs than those challenged with ICIPE 18 and ICIPE 665. The lowest hemocyte concentration was observed on the tenth-day post-infection among all the tested *M. anisopliae* isolates. Notably, *P. absoluta* moths infected with ICIPE 20 showed the most significant decline in THCs, followed by those challenged with ICIPE 18 and ICIPE 665 (67.53, 49.52, and 7.76%, respectively, relative to control) (Figure 2C; Supplementary Figure S4C).

**Figure 3:**
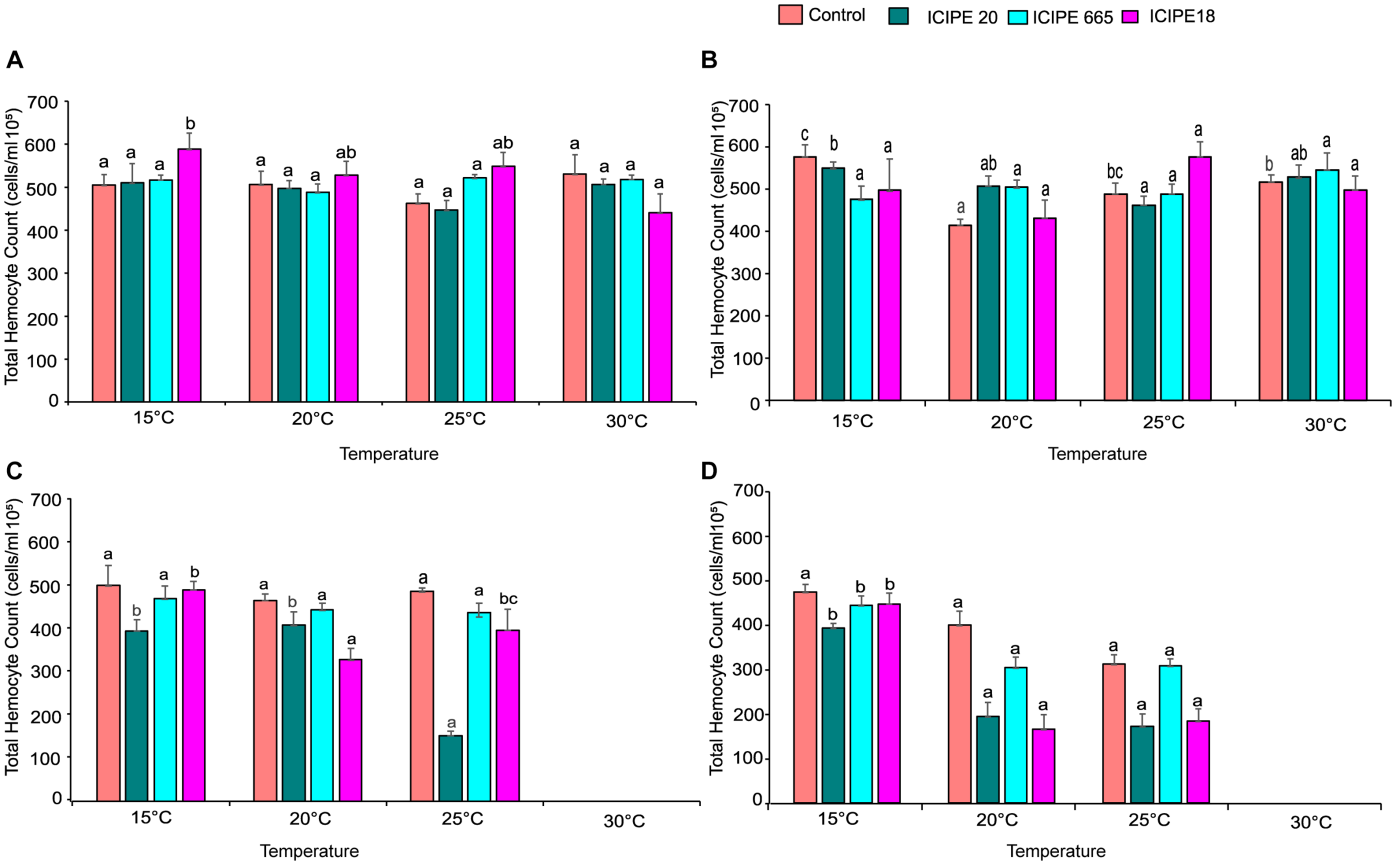
Effect of *Metarhizium. anisopliae* isolates and temperature on THC of *Phthorimaea absoluta* adults, **(A)** day 0, **(B)** day 1, **(C)** day 5, and **(D)** day 10 post-fungus-challenge. Different letters denote the significant difference (GLM, *p* ≤ 0.05).

In addition, we evaluated the combined/interactive impact of the tested *M. anisopliae* isolates and temperatures on THCs of *P. absoluta* adults at different intervals (i.e., days 0, 1, 5, and 10). On day 0 (2 h post-infection), we observed significant differences in THCs between fungus-challenged *P. absoluta* moths and their control counterparts at 15, 20, 25, and 30°C (χ^2^ = 23.18, df = 15 *p* = 0.03) (Figure 3A; Supplementary Figure S5A).

On day 1, after infecting the *P. absoluta* adults with the *M. anisopliae* isolates, there was no statistically significant difference in THCs between the challenged and control groups (χ^2^ = 20.25, df = 15, *p* = 0.16). However, it was noteworthy that the number of circulating hemocytes in the infected insect group was relatively lower than that of the control groups across the different temperature regimes (Figure 3B; Supplementary Figure S5B).

On day 5 after the fungus challenge, *P. absoluta* moths exhibited a significantly reduced number of THCs than the control counterparts (χ^2^ = 82.84, df = 10, *p* < 0.001). Notably, ICIPE 20-infected *P. absoluta* moths recorded the least circulating hemocytes at 15, 20, and 25°C. At 30°C, the temperature was fatal for the insects; thus, no data was recorded at this temperature regime (Figure 3C; Supplementary Figure S5C).

Finally, on day 10 post fungus challenge, the challenged *P. absoluta* adults recorded a significantly lower number of THCs than their control counterparts (χ^2^ = 39.13, df = 10, *p* < 0.001). ICIPE 20 infected *P. absoluta* adults had the least circulating hemocytes at 15, 20, and 25°C. No data was collected at 30°C since all insects (both challenged and controlled) were dead by 4th-day post-infection (Figure 3D; Supplementary Figure S5D). The phenoloxidase activity in the hemolymph of *P. absoluta* adults challenged with icipe 20 was higher than the control, while the PO activity of ICIPE 18 was lower than in the control insects at 24 h after infection (Supplementary Figure S6A). Furthermore, the daily PO activity in the hemolymph showed a declining pattern in the EPF-challenged treatments over time (Supplementary Figure S6B).

### 3.3. Effect of *Metarhizium anisopliae* on different hemocytes counts

**Figure 4:**
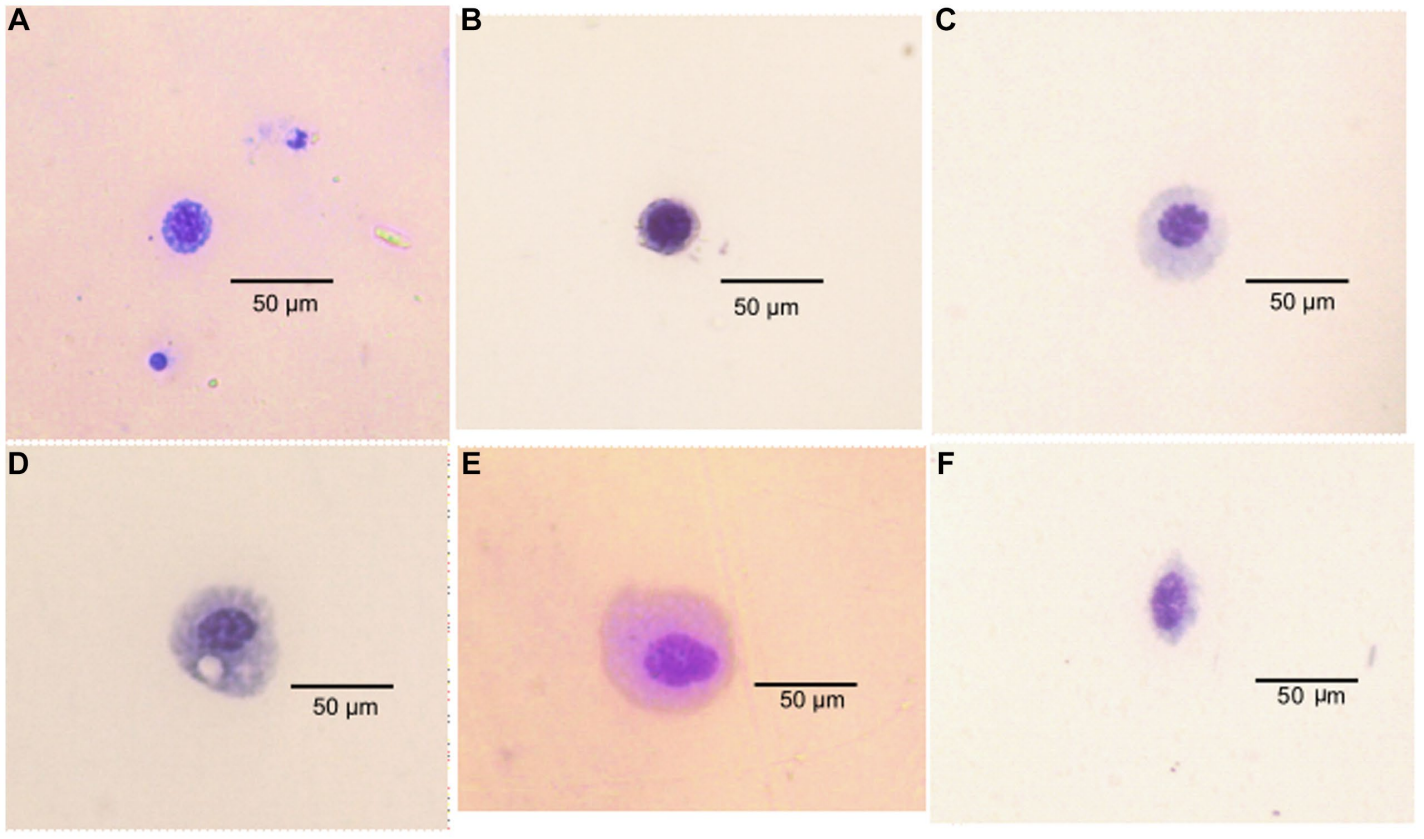
Types of hemocyte cells in *Phthorimaea absoluta* adults **(A)** granulocyte, **(B)** prohemocyte, **(C)** plasmatocytes, **(D)** adipohemocyte **(E)** adipohemocyte **(F)** spherulocyte.

Classification of the cells identified from *P. absoluta* hemolymph revealed six hemocyte types. The hemocytes were classified into six morphotypes, including Granulocytes (GR), Plasmatocytes (PL), Prohemocytes (PR), Oenocytoids (OE), Spherulocytes (SP), and Adipohemocytes (AD). Granulocytes appeared large and round and measured 25–30 μm. The nucleus was characterized by numerous small granules in the cytoplasm and a rounded, centrally placed nucleus. They were present in infected and uninfected host insects (Figure 4A).

The plasmatocytes were observed to have an elongated or oval shape with a 20–30 μm diameter and circular nuclei at the center. Their cytoplasm contained fine granules and vacuoles. They were present in *P. absoluta* moths of both challenged and control groups (Figure 4B). The prohemocytes were observed as small, round cells (with a diameter of 5–12 μm) that contained a centrally located purple-stained nucleus. They had a high nucleus-to-cytoplasm ratio and were the smallest among the identified hemocyte types in the challenged and control groups. They were present in both infected and control groups of *T. absoluta* moths (Figure 4C).

Oenocytoids were the biggest of all the hemocytes identified in *P. absoluta* adults, with a 30–40 μm diameter. Their cytoplasm appeared smooth and homogeneous, and their nucleus was eccentrically located. These hemocytes were round with a small central nucleus. They were present in fungus-challenged and unchallenged *P. absoluta* moths (Figure 4D). Spherulocytes appeared as spherical or oval-shaped cells of 17–25 μm diameter. Their cytoplasm was characterized by spherules and granules and had small and centrally placed nuclei. They were present in both infected and control groups of *P. absoluta* (Figure 4E). Adipohemocytes appeared as large oval cells of 15–20 μm diameter and had a centrally placed nucleus. After staining, the cytoplasm showed high basophilic and lipid-like inclusions. They were in fungus-challenged and unchallenged *P. absoluta* moths (Figure 4F). Among the identified hemocyte types, granulocytes were the most dominant (43–58%), followed by plasmatocytes (23–30%) in both fungus*-*challenged and control groups of *P. absoluta* adults at 15, 20, and 25°C. The other hemocyte types were present at low numbers, constituting 5–15% of the THC.

Analysis of differential hemocyte counts (DHCs) in *P. absoluta* adults in bioassays conducted at 15°C, specifically on day 0 (2 h after infection), showed a significant difference in oenocytoids compared to the control group (χ^2^ = 185.9, z = 2.44, *p* = 0.0147). Among the *P. absoluta* adults, those infected with ICIPE 665 exhibited the highest number of oenocytoids, while the group challenged with ICIPE 20 recorded the lowest number compared to the control group. On day 1 after fungus challenge, there were significant differences observed in the counts of granulocytes, plasmatocytes, and prohemocytes compared to the control groups (GRs: χ^2^ = 201.74, z = 2.46, *p* = 0.0141; PLs: χ^2^ = 154.09, z = 2.45, *p* = 0.0142; PRs: χ^2^ = 74.96, z = 2.46, 0.014 respectively). On day 5 post fungus challenge, plasmatocyte counts in the treatment groups were significantly lower than in the control groups (χ^2^ = 75.77, z = 2.45, *p* = 0.014). On day 10 after fungal infection, a significant difference was observed in the number of spherulocytes between the treatment and control groups (χ^2^ = 151.13, z = 2.44 *p* = 0.01). Among the *P. absoluta* moths, those infected with ICIPE 665 had the highest number of spherulocytes, whereas those infected with ICIPE 18 had the lowest counts. Although significant differences were observed between the treatment and control groups, no trend was observed in the DHCs across the time intervals (Supplementary Table S1).

In bioassays conducted at 20°C, the analysis of DHCs on day 0 showed a significant difference in counts of granulocytes, plasmatocytes, and prohemocytes between fungus infected and control groups (GRs: χ^2^ = 236.48, z = 2.45, *p* = 0.0141; PLs: χ^2^ = 140.65, z = 2.45, *p* = 0.014; PRs: χ^2^ = 166.44, z = 2.45, *p* = 0.0142 respectively). On day 1 after *M. anisopliae* infection, a significant difference was observed in the number of spherulocytes between the treatment groups and the control counterparts (χ^2^ = 160.02, z = 2.43, *p* = 0.015). Among the *P. absoluta* moths, those challenged with ICIPE 20 recorded the highest number of spherulocytes, whereas those infected with ICIPE 18 had the least spherulocytes. Also, on day 5 after the fungus challenge, the number of prohemocytes in the treatments was significantly lower than in the control groups (χ^2^ = 147.95, z = 2.45, *p* = 0.014). At the same time, the counts of plasmatocytes significantly differed between the treatment groups and control (χ^2^ = 93.84, z = 2.45, *p* = 0.0142). Finally, on day 10 after infection, the analysis of DHCs showed a significant difference between the number of adipohemocytes in the challenged groups and the control groups (χ^2^ = 615.3, z = 2.45, *p* = 0.014). Adult *P. absoluta* infected with ICIPE 20 and ICIPE 18 recorded the highest number of adipohemocytes, while those challenged with ICIPE 665 had the lowest adipohemocytes. While there were notable differences in DHCs between the treatments and control groups, there was no clear pattern across the time intervals (Supplementary Table S2).

Similarly, the variation in DHCs between fungal-infected and control adult *P. absoluta* moths was also observed in bioassays conducted at 25°C. On day 0, after exposing the insects at this specific temperature, there were significant differences observed in the number of granulocytes, plasmatocytes, prohemocytes, oenocytoids, and adipohemocytes between the treatment groups and the control groups (GRs: χ^2^ = 275.8, z = 2.45, *p* = 0.014; PLs: χ^2^ = 157.23, z = 2.45, *p* = 0.014; PRs: χ^2^ = 205.85, z = 2.45, *p* = 0.014; OEs: χ^2^ = 154.83, z = 2.42, *p* = 0.0154; ADs: χ^2^ = 105.3, z = 2.415, *p* = 0.0157). On days 1 and 5, the DHCs in the *M. anisopliae*–infected *P. absoluta* adult groups were not significantly different from the control groups. However, on day 10 post-infection, the number of plasmatocytes, prohemocytes, and spherulocytes in the treatment groups significantly differed from that of unchallenged moths (PLs: χ^2^ = 112.26, z = 2.46, *p* = 0.014; PRs: χ^2^ = 348.2, z = 2.45, *p* = 0.014; SP: χ^2^ = 195.79, z = 2.43, *p* = 0.015). Although significant variations in DHCs (dependent variables) were observed between the treatments and control groups, no clear trend could be identified across the different periods (Supplementary Table S3).

### 3.4. Microbiome community diversity

#### 3.4.1. Bacterial abundance composition

A total of 1,025,790 base-called reads with a base-pass rate of 65% were generated using two Nanopore MinION cells. Analysis of the 16S rRNA generated reads revealed. Firmicutes, Proteobacteria, Bacteroidetes, and Actinobacteria were identified as the most abundant bacterial phyla. All bacterial genera with a relative abundance of 0.1% and above were characterized, while those with a relative abundance of less than (<) 0.1% were collapsed into “others” in a stacked bar chart (Supplementary Figure S7).

**Figure 5:**
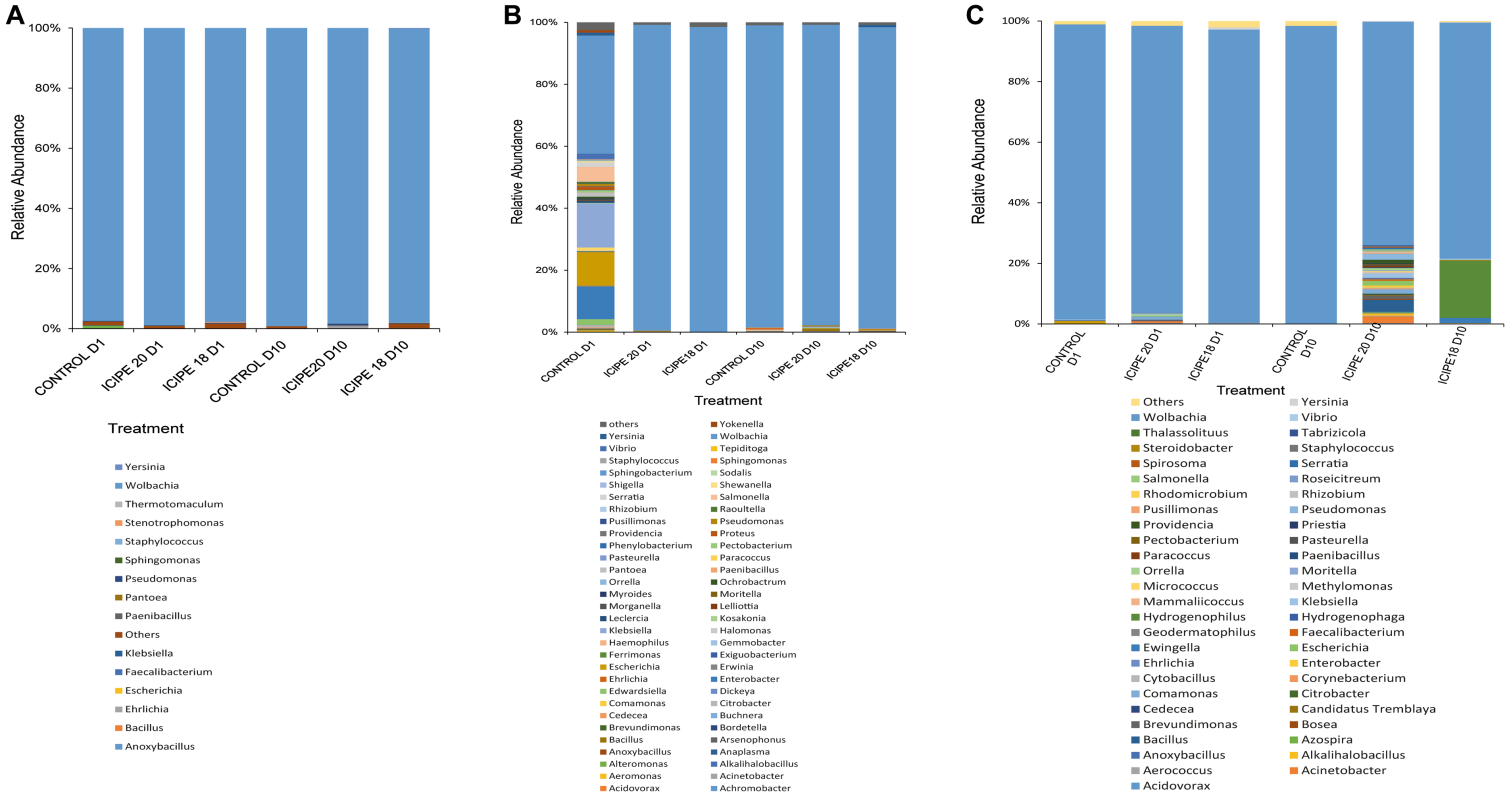
Relative abundance of bacterial communities in the gut of *Phthorimaea absoluta* adults at genus level after challenge with *Metarhizium anisopliae* ICIPE 18 and ICIPE 20 and subsequent incubation at **(A)** 15°C, **(B)** 20°C, and **(C)** 25°C. Data were analyzed on day one and day ten post-infection.

The analysis of relative cumulative abundance at the genus level revealed that *Wolbachia* was the most abundant bacterium in both fungus-challenged *P. absoluta* and their respective control groups. However, exposure of *P. absoluta* to *M. anisopliae* isolates at different temperature regimes led to significant changes in bacterial diversity. *P. absoluta* samples from bioassays conducted at 15°C had 15 bacteria genera (Figure 5A). In contrast, *P. absoluta* samples from bioassays conducted at 20°C exhibited 61 bacteria genera (Figure 5B). Similarly, 51 bacteria genera were obtained in *P. absoluta* samples from bioassays conducted at 25°C (Figure 5C). In addition, notable microbial shifts were observed from adult *P. absoluta* samples collected at 25°C on the tenth day after infection with ICIPE 20 and ICIPE 18. The relative abundance of *Wolbachia* declined by 27.68 and 20.94% in ICIPE 20 and ICIPE 18-infected *P. absoluta* adults, respectively, compared to the control group (Figure 5C).

#### 3.4.2. Alpha diversity

**Figure 6:**
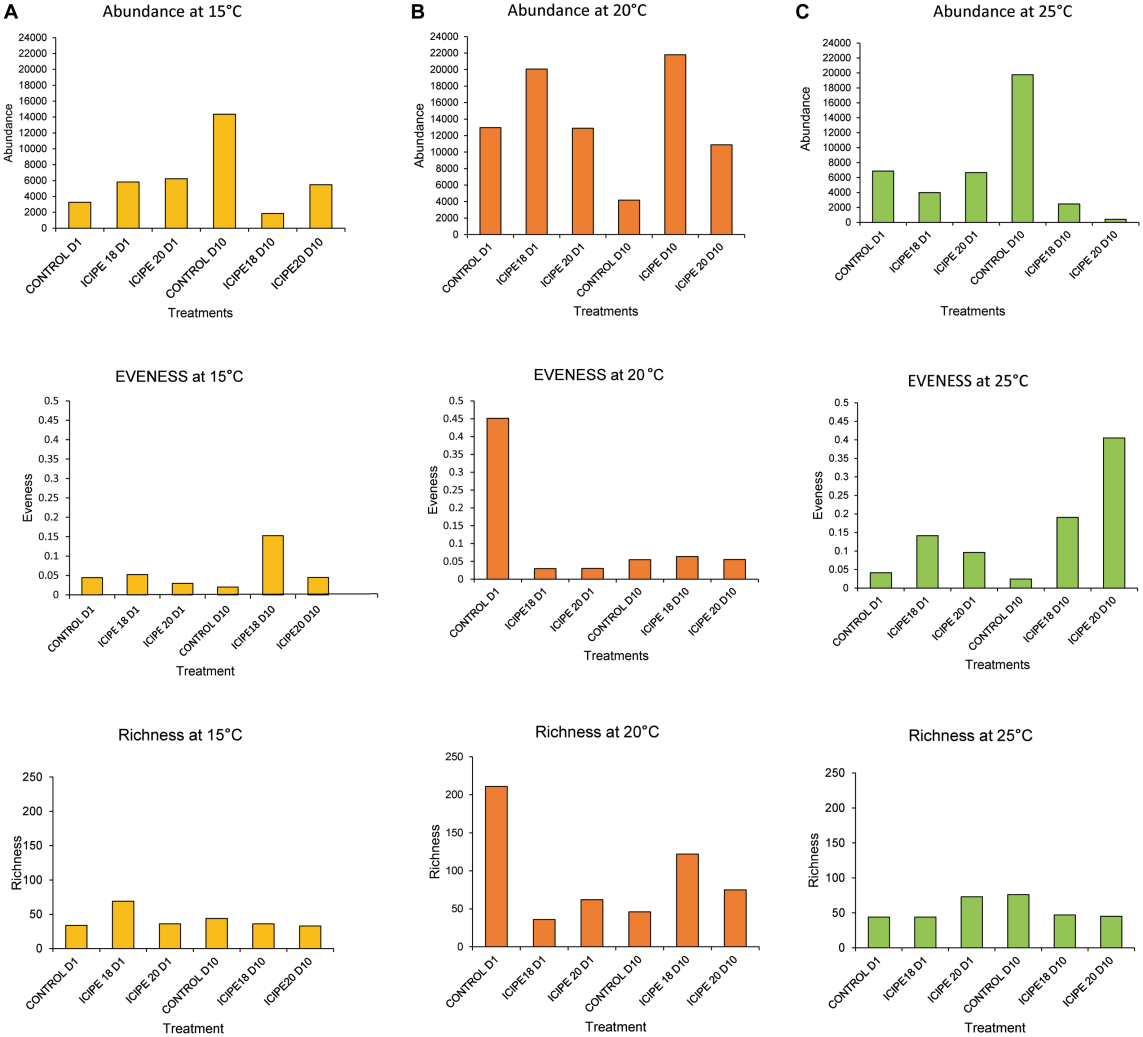
Abundance, evenness, and richness indices of bacterial communities of *Phthorimaea absoluta* adults challenged with *Metarhizium anisopliae* isolates ICIPE 18 and ICIPE 20 at 15°C **(A)**, 20°C **(B)**, and 25°C **(C)** on days one and ten post-treatment.

The alpha diversity indices of the microbiome were estimated using three metrics: Shannon, richness, and evenness. In bioassays conducted at 15°C, the Shannon diversity index showed that *P. absoluta* adults infected with ICIPE 18 had the highest microbial diversity (1.73). At the same time, ICIPE 20-infected insects recorded a microbial diversity of 1.17 on day 10 post-infection (Figure 6A). *P. absoluta* moths infected with ICIPE 18 and ICPE 20 at 20°C had a microbial diversity of 1.36 and 1.27, respectively, on day 10 after the fungus challenge (Figure 6B). Similarly, adult *P. absoluta* infected with ICIPE 20 at 25°C on day 10 had the highest microbial diversity (4.68), while those challenged with ICIPE 18 had a microbial diversity of 2.08 (Figure 6C).

Richness reflects a sample’s total number of bacteria (Young and Schmidt, 2008). In bioassays conducted at 15°C, ICIPE 18-infected *P. absoluta* adults exhibited a higher microbe richness index (36) than their counterparts challenged with ICIPE 20 (33) on day 10 post-infection (Figure 6A). Similarly, *P. absoluta* moths infected with ICIPE 18 at 20°C had a higher species richness index (122) than those infected with ICIPE 20 (75) on day 10 post-infection (Figure 6B). Finally, the species richness index for ICIPE18-infected *P. absoluta* adults was 47, while for those challenged with ICIPE 20 was 45 at 25°C on day 10 (Figure 6C).

Pielou’s evenness index showed that, in bioassays conducted at 15°C, ICIPE 18-infected *P. absoluta* moths exhibited a higher species distribution (0.15) than those infected with ICIPE 20 (0.04) on day 10 post-infection (Figure 6A). At 20°C, *P. absoluta* moths infected with ICIPE 18 had a higher microbe distribution (0.06) than their counterparts infected with ICIPE 20 (0.05) on day 10 after the fungal infection (Figure 6B). Finally, at 25°C, *P. absoluta* adults infected with ICIPE 20 had a higher evenness index (0.40) than those challenged with ICIPE 18 (0.19) on day 10 post-infection (Figure 6C).

#### 3.4.3. Beta diversity

**Figure 7:**
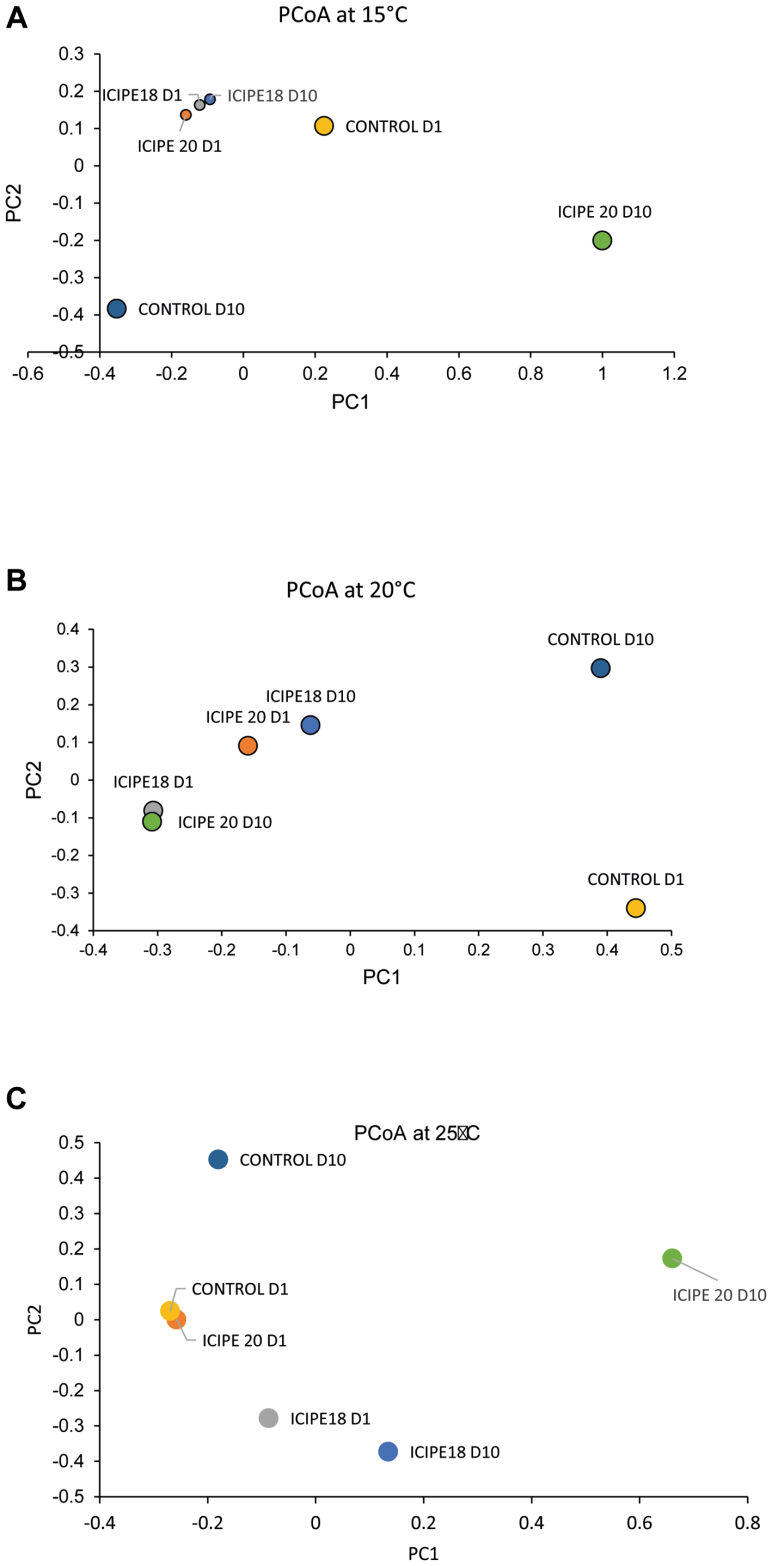
Two-dimensional principal coordinate plots of beta diversity of *Phthorimaea absoluta* microbiota challenged with *Metarhizium anisopliae* isolates ICIPE 18 and ICIPE 20 at **(A)** 15°C, **(B)** 20°C, and **(C)** 25°C on days one and ten post-treatment.

The effect of the fungal infection on the diversity of the gut bacterial community of *P. absoluta* adults was also estimated using the Jaccard dissimilarity index. Principal coordinate analysis (PCoA) showed a distinct clustering between bacterial communities in fungus-challenged insects and control (untreated) insects. In *P. absoluta* moths incubated at 15°C, the highest microbial diversity was recorded between ICIPE 20-infected insects and their control counterparts (88.68%) on day 10. However, the lowest microbe diversity was recorded between ICIPE 18-infected *P. absoluta* adults on days 1 and 10 (8.44%) (Figure 7A; Supplementary Table S4).

In bioassays conducted at 20°C, the highest microbial diversity was recorded between ICIPE 20-infected moths on day 10 and control counterparts on day 1 (81.57%). At the same time, the lowest bacterial diversity was observed between ICIPE 20-infected *P. absoluta* adults on day 10 and moths challenged with ICIPE 18 on day 1 (8.47%) (Figure 7B; Supplementary Table S5).

Finally, in adult *P. absoluta* adults incubated at 25°C, the highest microbial diversity was observed between ICIPE 20-infected moths and control counterparts on day 10 (98.03%). At the same time, the lowest species diversity was recorded between ICIPE 20-challenged *P. absoluta* moths and unchallenged insects on day 1 (11.08%) (Figure 7C; Supplementary Table S6).

### 3.5. Correlation between survival rates and immune responses of *Phthorimaea absoluta* post-*Metarhizium anisopliae* exposure

**Figure 8:**
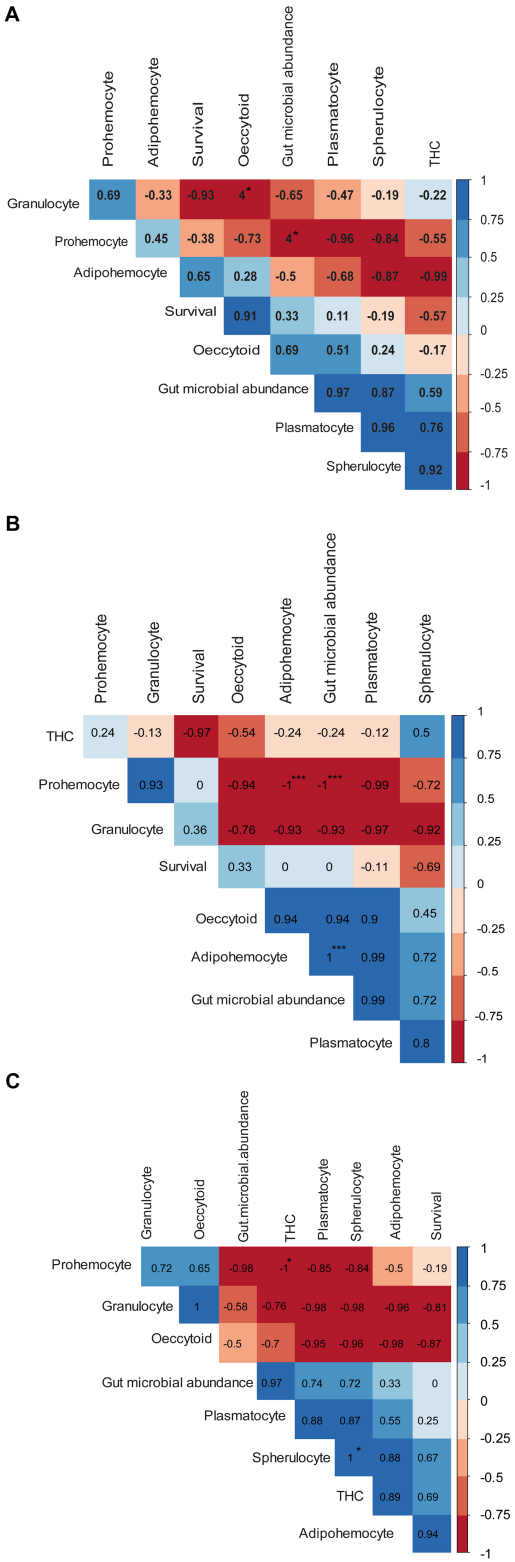
Correlograms showing the relationship between survival, total hemocyte counts, differential hemocyte counts, and gut microbial abundance when *Phthorimaea absoluta* is exposed to *Metarhizium anisopliae* under different temperature regimes **(A)** 15°C, **(B)** 20°C and **(C)** 25°C. Asterisks represent significant correlation (** for *p* < 0.01 and * for *p* < 0.05). The Pearson correlation coefficients (r) for each pair of compared parameters are represented by the numbers inside each box.

Pearson’s correlation analysis showed that at 15°C, prohemocyte counts were negatively correlated with plasmatocytes and spherulocytes (Figure 8A). On the other hand, THCs, spherulocytes, and plasmatocytes were positively correlated, while survival rate was positively correlated to oenocytoid counts. At 20°C, the THCs were negatively correlated with the insect survival rate of *P. absoluta* (Figure 8B). On the other hand, plasmatocyte counts were positively correlated with gut microbial abundance, adipohemocytes, and oenocytoids. In addition, prohemocyte counts were positively correlated with granulocyte counts. Finally, at 25°C, prohemocyte counts were negatively correlated with gut microbial abundance, THCs, plasmatocytes, and spherulocytes (Figure 8C). On the other hand, spherulocyte counts and plasmatocytes, THCs, and gut microbial abundance were positively correlated between granulocytes and oenocytoids.

## 4. Discussion

Entomopathogenic fungi control insects by infecting and killing them through complex mechanisms (Migiro et al., 2010; Jiang et al., 2020). When the spores of these fungi come into contact with the host insect, they can attach to the cuticle and germinate, penetrating the insect’s body through the cuticle, mouthparts, or spiracles (Wang et al., 2021). Once inside the insect, the fungi grow and produce enzymes and toxins that break down the insect’s tissues and organs, ultimately leading to death (Skinner et al., 2014). However, the effectiveness of EPF depends on several factors, such as the fungal species, the target host insect, and the environmental conditions (Bugeme et al., 2008; Shamakhi et al., 2019). Our research offers new insights into EPF infections and interactions with the innate immune responses and gut microbiome dynamism of *P. absoluta*. The cellular immune response and gut immunity protect insects against infections with entomopathogenic filamentous fungi (Yassine et al., 2012; Ramirez et al., 2019).

When *P. absoluta* moths were exposed to dry *M. anisopliae* conidia, their survival rate gradually declined as the temperature increased from 15 to 30°C. After infection, all three fungal isolates induced the highest mortality rate at 25°C, which seemed to be the optimal temperature for the maximum impact of these fungal strains’ infection on the pest. Adult *P. absoluta* challenged with ICIPE 18 had the lowest survival rate, followed closely by those infected with ICIPE 20, while moths challenged with ICIPE 665 showed the highest survival rate. Our findings align with earlier research studies reported by Akutse et al. (2020a,b) and Agbessenou et al. (2021), who demonstrated the substantial potential of *M. anisopliae* isolates ICIPE 18 and ICIPE 20 against adult *P. absoluta*. Onsongo et al. (2019) also reported comparable results, where *M. anisopliae* isolates, ICIPE 18 and ICIPE 69 caused the most significant mortality in adult Tephritid fruit flies (*Zeugodacus cucurbitae*) at 25°C. Tumuhaise et al. (2015) conducted a study on temperature-dependent effects, which revealed that the most significant mortality rates were observed between 25 and 30°C when combating adult legume pod borer (*Maruca vitrata* Fabricius) using *M. anisopliae* isolate ICIPE 69. The rapid germination of the *M. anisopliae* isolates at 25°C could probably explain the fastest/highest mortality they induced in the host insect pests. This phenomenon confirms that fungal-based insecticides kill insect pests most rapidly at the optimum temperature of their vegetative growth (Tumuhaise et al., 2015). Our findings demonstrate that ICIPE 20 and ICIPE 18 could be deployed in ecological areas with temperatures around 25°C for sustainable management of adult *P. absoluta, which* has also been reported by Agbessenou et al. (2021). Since the efficacy of EPF against insect pests is temperature-dependent, the virulence pattern observed in this study is crucial in choosing key application areas for these biopesticides.

When EPF penetrate the body of insect pests, they activate both cellular and humoral immune responses that work to protect the insect host from the invading pathogens (Wang J.-L. et al., 2022). In response, the infected insects release more hemocytes to fight the external stimuli (Shamakhi et al., 2019). These hemocytes play a critical role in mediating cellular immune responses, which are important in helping insects fight against invading pathogens (Stoepler et al., 2013). In the process, hemocytes may adhere to the surface of the fungal hyphae, which can reduce their invasion and virulence to defend against the infection.

In this study, we observed a significant reduction in the total hemocyte counts (THCs) in *P. absoluta* adults exposed to *M. anisopliae* isolates, with the decline increasing steadily from 15°C to 25°C. The declining pattern in the concentration of THCs could be linked to the ability of the tested *M. anisopliae* isolates to produce toxins that affect the hemocytes’ viability or function/virulence. In addition, the immune response to the fungal infection may have led to the depletion of hemocytes, as they are recruited and consumed by the host insect to fight the infection. Freitas et al. (2015) also witnessed a comparable reduction in the number of hemocytes 48 h after infecting *Rhipicephalus microplus* ticks with *Beauveria bassiana* CG 206 conidia. Additionally, Zhong et al. (2017) reported a decrease in THCs following infection of the cotton bollworm *Helicoverpa armigera* (Hübner) (Lepidoptera: Noctuidae) with *M. anisopliae.* In contrast, Mishra et al. (2015) noted an opposite effect: the THCs increased in housefly larvae *Musca domestica* Linnaeus (Diptera: Muscidae) after exposure to *B. bassiana*.

Our findings also demonstrated significant post-infection alterations in the constitutive hemocyte types in host insects. For instance, variations in the granulocyte counts after the fungus challenge, may indicate fungus-induced differentiation and secretion of granulocytes into the hemolymph. However, a decline in plasmatocyte density was observed in *P. absoluta* infected with fungus. The observed pattern of granulocyte increase and plasmatocyte decrease following *M. anisopliae* infection may be linked to increased granulocyte differentiation and selective plasmatocyte apoptosis. Among the differential hemocyte counts observed in this study, the specific roles of spherulocytes and adipohemocytes in innate immunity are unknown (Altuntaş et al., 2021; Gwokyalya et al., 2022). However, oenocytoids have been stated to play an essential role in mediating melanization by activating the phenol oxidase cascade (Gwokyalya et al., 2022). Therefore, the increase in oenocytoids following fungal infection may be due to the host’s response to stimulate the phenoloxidase melanization of the fungal germ tubes and consequently block the pathogen activity within the host. Although *M. anisopliae* conidia is easily transmitted to the pest through contact, the duration of infection is relatively long (24–72 h) (Sinha et al., 2016). This explains the variations in hemocyte types observed on day 0 (2 h post-infection) that may be due to other factors such as temperature changes.

The decrease in circulating hemocytes following infection led us to investigate the microbial community in the gut of the infected *P. absoluta* adults, as the fungal infection could directly impact the gut microbiome. Fungal infections can disrupt gut bacterial diversity by altering the microbial balance, causing inflammation, and affecting the gut environment (Wei et al., 2017). This disruption can lead to imbalances in the microbiota, impacting digestion, immune responses, and overall gut health (Siddiqui et al., 2022). Our results showed an upsurge in bacterial load in infected *P. absoluta* moths on the tenth-day post-infection. The level of microbiome loads increase appeared to reflect the magnitude of the immune response since *P. absoluta* adults that had recorded the highest THCs decline (ICIPE 20 and ICIPE 18-infected *P. absoluta*) also had the highest upsurge in microbiota diversity at 25°C. The effect of EPF on the gut microbiome was previously examined by Wei et al. (2017) in *Anopheles stephensi* Liston (Diptera: Culicidae) mosquitoes after infection with *B. bassiana*. The study demonstrated an increase in gut bacterial loads in mosquitoes. In addition, the authors found that increased bacterial loads led to accelerated deaths of infected mosquitoes. Therefore, the rise in gut bacterial load is most likely due to the indirect result of the multifaceted response mounted by *P. absoluta* against the fungal infection challenges/stresses. A microbiome analysis between the treatment groups showed no significant microbial shifts in the gut bacterial composition in *P. absoluta* adults incubated at 15 and 20°C. The slow mode of action of *M. anisopliae* infection at these temperature regimes could cause an unnoticeable change in bacterial abundances in *P. absoluta*. However, *M. anisopliae* infection caused significant microbial shifts at 25°C. The variations in the relative abundance of *Wolbachia* to other bacterial genera, including *Hydrogenophilus*, *Ewingella Bacillus, Klebsiella*, *Escherichia*, *Sphigomonas*, *Pseudomonas*, *Ewingella, Vibrio,* and *Enterobacter,* highly suggest that this endosymbiont might play an essential role in the course of fungal infection. Wang J.-L. et al. (2022b) observed a similar gut microbial shift pattern following topical fungal infection of brown planthopper, *Nilaparvata lugens* (Stål) (Hemiptera: Delphacini) with *M. anisopliae*.

*Wolbachia* was the predominant bacterial genera in infected *P. absoluta* groups and their control counterparts (control groups) across all the temperature regimes. It is a crucial endosymbiont in many insects (Gichuhi et al., 2020). *Wolbachia* has been proposed as a potential component that can be explored to develop control approaches against host insects (Rancès et al., 2012). Studies show that *Wolbachia* induces host reproductive manipulation, including cytoplasmic incompatibility, feminization, male killing, and parthenogenesis (Oliver et al., 2022). In adult *P. absoluta* infected with ICIPE 18 and incubated at 25°C, we noted an upsurge of *Ewingella, Vibrio,* and *Hydrogenophilus*. In addition, at the same temperature regime, we noted an upsurge in *Acinetobacter, Bacillus, Klebsiella, Providencia, Pseudomonas, Staphylococcus,* and *Serratia* in ICIPE 20-infected *P. absoluta*. *Ewingella* and *Vibrio* are pathogenic gram-positive bacteria that have been reported to cause high mortality in insects and humans (Meisler et al., 2020). *Acinetobacter* is a bacterial genus found in insect gut that provides the host with nutrients and insecticidal potential that could be pathogenic against their host (Wang et al., 2022a). *Bacillus* is an entomopathogenic bacteria used for microbial pest management on various insect pests, including *Spodoptera frugiperda* (Lepidoptera: Noctuidae) (Ruiu, 2015). Other bacteria showing pathogenic potential against insect pests include *Serratia, Providencia,* and *Pseudomonas* (Ruiu, 2015; Deka et al., 2021). As far as we know, no research has been conducted to investigate the pathogenic effects of *Hydrogenophilus* on *P. absoluta*.

Our results indicated that at 25°C, *P. absoluta* adults infected with ICIPE 20 had the highest evenness index, while those challenged with ICIPE 18 exhibited the greatest microbial diversity. These findings suggest that ICIPE 20 caused all bacterial communities within *P. absoluta* to have a higher expression rate of equal abundance within the microbiome than the insects challenged with ICIPE 18. Analysis of species richness showed that *P. absoluta* challenged with ICIPE 20 had the most bacterial taxa and, thus, were likely to have a more complex microbiota. The species evenness and richness in fungus-challenged *P. absoluta* were positively correlated. Ajene et al. (2020) reported that species’ evenness and richness positively correlate within invertebrate communities. The Jaccard Dissimilarity indices showed significantly different clustering of fungus-challenged *P. absoluta* moths and uninfected insects. This suggests that the bacterial community structure in *M. anisopliae*-infected *P. absoluta* differed from that of their control counterparts. Overall, the alpha and beta diversity of the metagenome of adult *P. absoluta* supports the hypothesis that infection with *M. anisopliae* disrupts gut bacterial community structure.

We also expected the survival rate, THCs, and gut microbial abundance to be positively correlated when *P. absoluta* was infected with ICIPE 20 at different temperature regimes (Onchuru and Kaltenpoth, 2019). However, this assumption held for prohemocytes, plasmatocytes, spherulocytes, THCs, plasmatocytes, and survival rate at 15°C. It seems likely that the high numbers of THCs are responsible for the defense of host insects, hence the higher survival. At 20°C, THCs were negatively correlated with survival rate, which might explain the decline in survival of *P. absoluta.* In addition, the positive correlation of plasmatocytes and gut microbial abundance might indicate increased immune response in the host-insect after *M. anisopliae* infection. A similar correlation was observed at 25°C, seemingly indicating hemocytes and gut microbiome are the primary defense mechanism used by *P. absoluta* when infected by *M. anisopliae*.

## 5. Conclusion

In conclusion, our study provides valuable insights into the potential of *M. anisopliae* isolates, specifically ICIPE 18 and ICIPE 20, as effective biocontrol agents against *P. absoluta* adults. The findings of this study underscore the significance of immunological changes and gut microbial shifts in influencing the survival and fitness of *P. absoluta* adults. The mortality induced by ICIPE 18 suggests strong pathogenicity, and ICIPE 20’s impact underscores its potential as a potent agent of insect pest population control. The significant reduction in THCs in response to these isolates points to the disruption of the insect’s immune response. This immune system suppression could render pests more susceptible to infections and environmental stressors. Moreover, the observed gut microbial shifts induced by both isolates at 25°C present a crucial mechanistic insight. Alteration in the gut microbiota can significantly affect insect digestion, nutrient utilization, and overall fitness. By weakening both immune defenses and gut health, ICIPE 18 and ICIPE 20 act synergistically to compromise the survival and vitality of *P. absoluta* adults. These findings collectively support the potential of ICIPE 18 and ICIPE 20 as biocontrol agents. Their ability to disrupt physiological processes, weaken immune responses, and induce gut microbial shifts suggests a multi-pronged approach to pest management. Future research could delve deeper into the mechanisms underlying these effects and further explore their long-term ecological implications to validate their role as sustainable alternatives to chemical insecticides.

## Data availability statement

The data presented in the study are deposited in the NCBI GenBank repository https://www.ncbi.nlm.nih.gov/genbank, Bioproject number PRJNA970552.

## Ethics statement

The manuscript presents research on animals that do not require ethical approval for their study.

## Author contributions

FM: Data curation, Formal analysis, Investigation, Methodology, Validation, Writing – original draft, Writing – review & editing. KA: Conceptualization, Data curation, Funding acquisition, Resources, Supervision, Validation, Writing – review & editing. IA: Conceptualization, Data curation, Formal analysis, Investigation, Methodology, Software, Validation, Visualization, Writing – review & editing. KO: Resources, Supervision, Writing – review & editing. FK: Conceptualization, Data curation, Funding acquisition, Project administration, Resources, Supervision, Writing – review & editing.
