# Supplementary material for: Immunological responses and gut microbial shifts in Phthorimaea absoluta exposed to Metarhizium anisopliae isolates under different temperature regimes
Source: Front Microbiol. 2023 Nov 9;14:1258662. doi: 10.3389/fmicb.2023.1258662 (PMC10666277; doi:10.3389/fmicb.2023.1258662)
Supplement: Supplementary file 1 [file Data_Sheet_1.pdf]

Supplementary Material

Supplementary Table 1: Effect of *Metarhizium anisopliae* strains on DHC at 15ºC. For each hemocyte type, means with different letters within a column across treatment groups in the same time interval denote a significant difference ($p \leq 0.05$, n=60)

Time	Isolate	Plasmatocytes	Prohemocytes	Granulocytes	Spherulocytes	Adipohemocytes	Oenocytoids
Day 0	Control	20.67± 1.76a	17.67± 1.20a	42.33±2.33a	6±1.73a	5.67±2.03a	7.67±0.88a
	ICIPE 20	21.67± 2.19a	19±2.52a	38±1.15a	13.67±2.40a	3±1.15a	4.67±0.88a
	ICIPE 18	21.33± 2.33a	19.67±2.19a	38±1.15a	12.67±2.33a	3.33±1.45a	5±1.15a
	ICIPE665	20.33±2.96 a	17±2.08a	38.33±2.33a	5.67±1.86a	7.67±2.19a	11±2.08b
Day 1	Control	14.67±1.85ab	27±2.08b	43.67±2.33a	7.3±1.20a	3.33±0.88a	4±0.58a
	ICIPE 20	19.33±2.19b	12.67±4.41a	46±3.21a	10.33±3.33a	7.3±2.84a	4.3±1.20a
	ICIPE 18	12.33±2.03ab	15±1.15ab	56±1.76b	4±1.53a	6.67±1.20a	5.33±2.19a
	ICIPE665	11.67±1.76a	22±3.21ab	39±2.30a	10±2.31a	6±0.58a	11.3±4.40a
Day 5	Control	13.33±3.18ab	19.67±0.88a	59.33±4.98a	3±1.53a	1.67±0.33a	3±0.58a
	ICIPE 20	23±3.46b	15±2.66a	51.67±2.02a	6±2.51a	1.6±0.88a	3±1.15a
	ICIPE 18	9.33±2.40a	20.67±2.91a	53±8.08a	6±1.53a	6.67±2.67a	6±1.20a

	ICIPE665	9.33±3.18a	19±1.53a	61.67±2.91a	2.3±0.88a	3.33±1.33a	4.33±1.33a
Day10	Control	19±3.46a	17.67±3.80a	46.33±4.67a	6±1.53ab	6.67±0.88a	4.33±1.20a
	ICIPE 20	14.67±2.96a	27.67±3.17a	42.33±1.76a	5.3±0.67ab	5±1.15a	5±2.08a
	ICIPE 18	22±2.65a	16.67±3.18a	46.67±2.91a	4±1.15a	4.33±2.03a	6.33±2.33a
	ICIPE665	17±2.08a	17±3.61a	46.67±4.81a	10±2.08b	5±1.53a	4.33±1.20a

Supplementary Table 2: Effect of *Metarhizium anisopliae* strains on DHC at 20ºC. For each hemocyte type, means with different letters within a column across treatment groups in the same time interval denote a significant difference ($p \leq 0.05$, n=60)

Time	Isolate	Plasmatocytes	Prohemocytes	Granulocytes	Spherulocytes	Adipohemocytes	Oenocytoids
Day 0	Control	17.67±2.73a	22.33± 2.40bc	42.67±1.45ab	5.67±2.91ab	6±0.58a	5.67±1.86a
	ICIPE 20	13.33±2.18a	26±2.64c	45±3.60ab	6.67±1.45ab	2.33±0.88a	6.67±1.20a
	ICIPE 18	20.67±2.19a	17±2.31ab	38.67±1.45a	6±1a	9±1.52a	8.67±0.88a
	ICIPE665	19.67±1.76a	13±1.15a	48.33±1.86b	6.33±0.88b	6±1.15a	6.67± 2.03a
Day 1	Control	16±4.04a	19±5.50a	45±3.60a	7±1.15ab	6.67±2.40a	6.33±0.88a
	ICIPE 20	12±0.58a	24.33±3.75a	45.33±2.33a	8±0.58b	5±2.08a	5.33±1.45a

	ICIPE 18	19±2.08a	17.33±1.76a	51±2.31a	2.33±1.45a	5.33±a	5±1.15a
	ICIPE665	19±2.08a	17.67±1.76a	44.33±3.38a	5.67±1.67ab	8±1.73a	5.33±1.45a
Day 5	Control	9.67±2.72a	25.33±3.38b	53± 7.23a	3.67±1.20a	4±0.88a	5.33±7.2a
	ICIPE 20	13±3.05ab	14.33±1.45a	55.33±4.48a	6±1.53a	7±0.58a	4.67±1.20a
	ICIPE 18	21.33±1.45b	19.33±1.86ab	48.33±2.33a	2.67±1.20a	5±0.88a	3.33±0.88a
	ICIPE665	15.67±2.40ab	17.33±2.33ab	52±4.40a	6±1.85a	4±0.58a	5±1.20a
Day10	Control	16 ±1.73a	21.67±1.85a	45.67±3.38a	7.33±1.67a	6.67±0.88b	2.67± 1.20a
	ICIPE 20	13.33±2.90a	19±4.04a	51.33±1.85a	6.33±1.20a	7±0.58b	3± 1.53a
	ICIPE 18	16±1.15a	17±2.52a	48.33±1.45a	6.33±1.20a	7±0.58b	3.67±1.20a
	ICIPE665	15.67±2.40a	17.33±2.19a	52±4.73a	6±1.73a	4±0.58a	5±1.15a

Supplementary Table 3: Effect of *Metarhizium anisopliae* strains on DHC at 20ºC. For each hemocyte type, means with different letters within a column across treatment groups in the same time interval denote a significant difference ($p \leq 0.05$, n=60)

Time		Plasmatocytes	Prohemocytes	Granulocytes	Spherulocytes	Adipohemocytes	Oenocytoids
Day 0	Control	12.33±2.73ab	23.33±0.88b	51.67±3.48b	6.33±1.20a	4.33±0.88ab	2±0.58a
	ICIPE 20	11.33±0.88a	25.33±2.84b	52±1.73b	4.67±1.33a	3±0.33a	3.67±0.88ab

	ICIPE 18	19.67±1.76c	18.33±2.03ab	39.33±0.88a	7.33±1.20a	8.33±0.58b	8±0.88b
	ICIPE665	18.67±1.76bc	14.67±1.76a	50.67±1.45b	6.33± 1.45a	4.33±1.76ab	5.33±1.76ab
Day 1	Control	10.33±3.38a	20.33±3.48a	55.33±5.36a	4.33±1.86a	4.67±1.45a	5±2.08a
	ICIPE 20	8.33±1.45a	25.67±3.17a	60.67±3.71a	3.33±0.33a	3.33±0.33a	3.33±0.88a
	ICIPE 18	19.33±1.76a	19±1.15a	46±2.52a	7±2.08a	4.33±2.40a	4.33±1.45a
	ICIPE665	15.33±4.91a	16.33±4.05a	50.33±6.12a	5±3.21a	5±0a	8±2.08a
Day 5	Control	18.33±1.85a	18.33±3.48a	45.67±1.45a	4.67±1.76a	7.33±2.91a	5.67±0.67a
	ICIPE 20	16±6.08a	14.67±4.48a	51.67±5.78a	4.33±0.88a	7.67±1.45a	5.67±1.86a
	ICIPE 18	21.67±1.76a	18.33± 1.86a	41.67±2.03a	5.33±0.88a	6.67±1.45a	6.33±0.88a
	ICIPE665	17.33±3.17a	15±5.56a	49.67±4.41a	6±3.51a	4.67±1.45a	7.33± 1.20a
Day10	Control	20.33±2.40ab	19.67±1.86ab	44.33±3.28a	6.33±1.20b	5.33±1.20a	4±2a
	ICIPE 20	12.33±2.73a	23.33±0.88ab	51.67±3.48a	6.33±1.20b	4.33±0.88a	2±0.58a
	ICIPE 18	25.67±3.18b	18.67±1.45a	41±2.30a	2±0.58a	4.33±0.88a	8.33±2.40a
	ICIPE665	19.33±1.20ab	25±1.73b	43±3.61a	6.33±1.20b	4.33±0.88a	2±0.58a

Supplementary Table 4: Beta diversity (%) in the metagenomes of *Phthorimaea absoluta* after treatment with *Metarhizium anisopliae* strains ICIPE 18 and ICIPE 20 at 15°C as estimated using the Jaccard dissimilarity index.

	**Dissimilarity (%)**					
**Treatments**	ICIPE 20 D1	ICIPE 18 D1	CONTROL D1	ICIPE 18 D10	ICIPE 20 D10	CONTROL D10
ICIPE 20 D1	-					
ICIPE 18 D1	10.03%	-				
CONTROL D1	48.56%	44.84%	-			
ICIPE 18 D10	14.72%	8.44%	41.53%	-		
ICIPE 20 D10	74.63%	72.41%	52.50%	71.15%	-	
CONTROL D10	57.19%	60.44%	77.56%	62.64%	88.68%	-

Supplementary Table 5*:* Beta diversity (%) in the metagenomes of *Phthorimaea absoluta* after treatment with *Metarhizium anisopliae* strains ICIPE 18 and ICIPE 20 at 20°C as estimated using the Jaccard dissimilarity index.

	**Dissimilarity (%)**					
**Treatments**	ICIPE 20 D1	ICIPE 18 D1	CONTROL D1	ICIPE 18 D10	ICIPE 20 D10	CONTROL D10
ICIPE 20 D1	-					
ICIPE 18 D1	36.62%	-				

CONTROL D1	75.25%	81.41%	-			
ICIPE 18 D10	19.21%	47.76%	72.29%	-		
ICIPE 20 D10	41.32%	8.47%	81.57%	51.34%	-	
CONTROL D10	68.01%	79.55%	68.07%	62.14%	80.98%	-

Supplementary Table 6: Beta diversity (%) in the metagenomes of *Phthorimaea absoluta* after treatment with *Metarhizium anisopliae* strains ICIPE 18 and ICIPE 20 at 25°C as estimated using the Jaccard dissimilarity index.

	**Dissimilarity (%)**					
**Treatments**	ICIPE 20 D1	ICIPE 18 D1	CONTROL D1	ICIPE 18 D10	ICIPE 20 D10	CONTROL D10
ICIPE 20 D1	-					
ICIPE 18 D1	51.47%	-				
CONTROL D1	11.08%	53.02%	-			
ICIPE 18 D10	72.97%	57.12%	73.86%	-		
ICIPE 20 D10	94.25%	91.80%	94.93%	88.07%	-	
CONTROL D10	67.84%	82.83%	65.43%	90.43%	98.03%	-

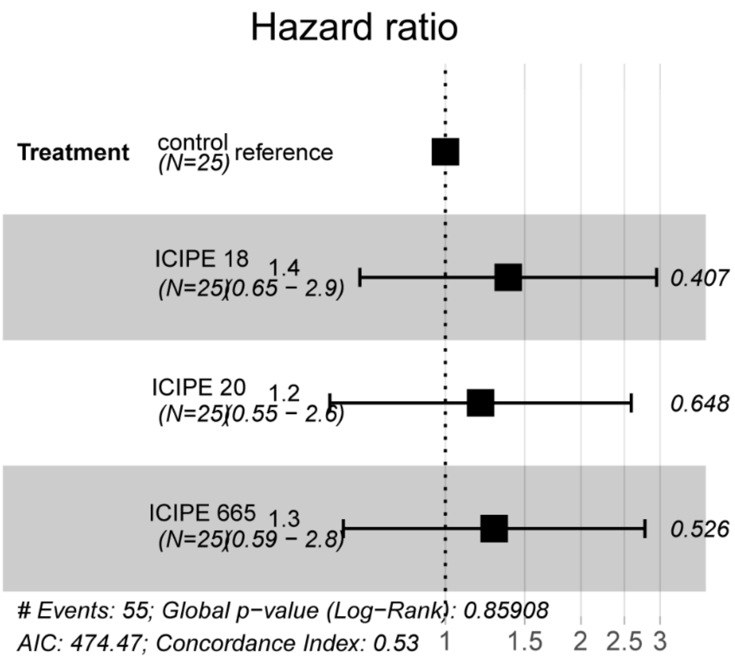

Supplementary Figure 1: Hazard ratios for the *Metarhizium anisopliae* isolates (ICIPE18, ICIPE 20, and ICIPE 665) at 15°C for 10 days (Cox proportional $p<0.05$).

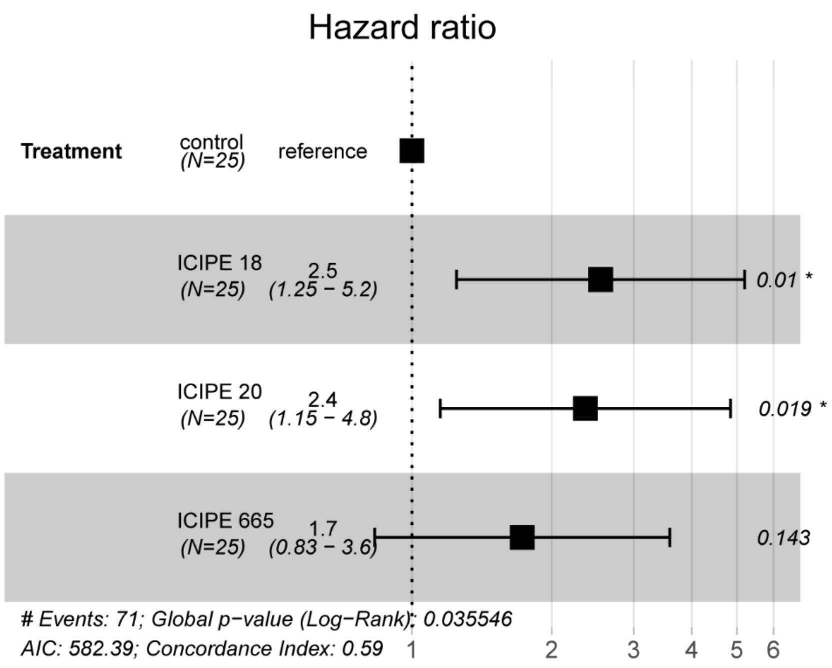

Supplementary Figure 2: Hazard ratios for the *Metarhizium anisopliae* isolates (ICIPE18, ICIPE 20, and ICIPE 665) at 20°C for 10 days (Cox proportional p<0.05).

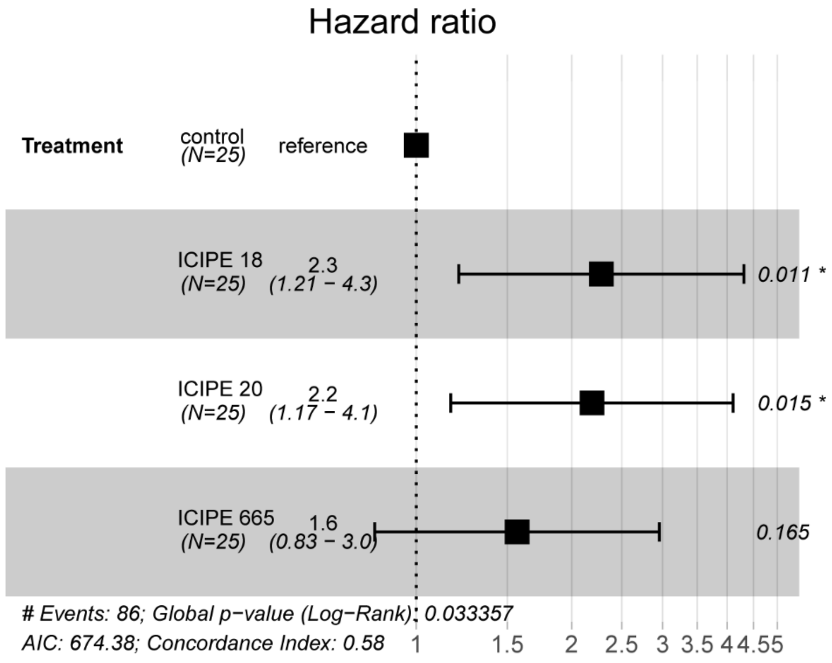

Supplementary Figure 3: Hazard ratios for the *Metarhizium anisopliae* isolates (ICIPE18, ICIPE 20, and ICIPE 665) at 20°C for 10 days (Cox proportional p<0.05).

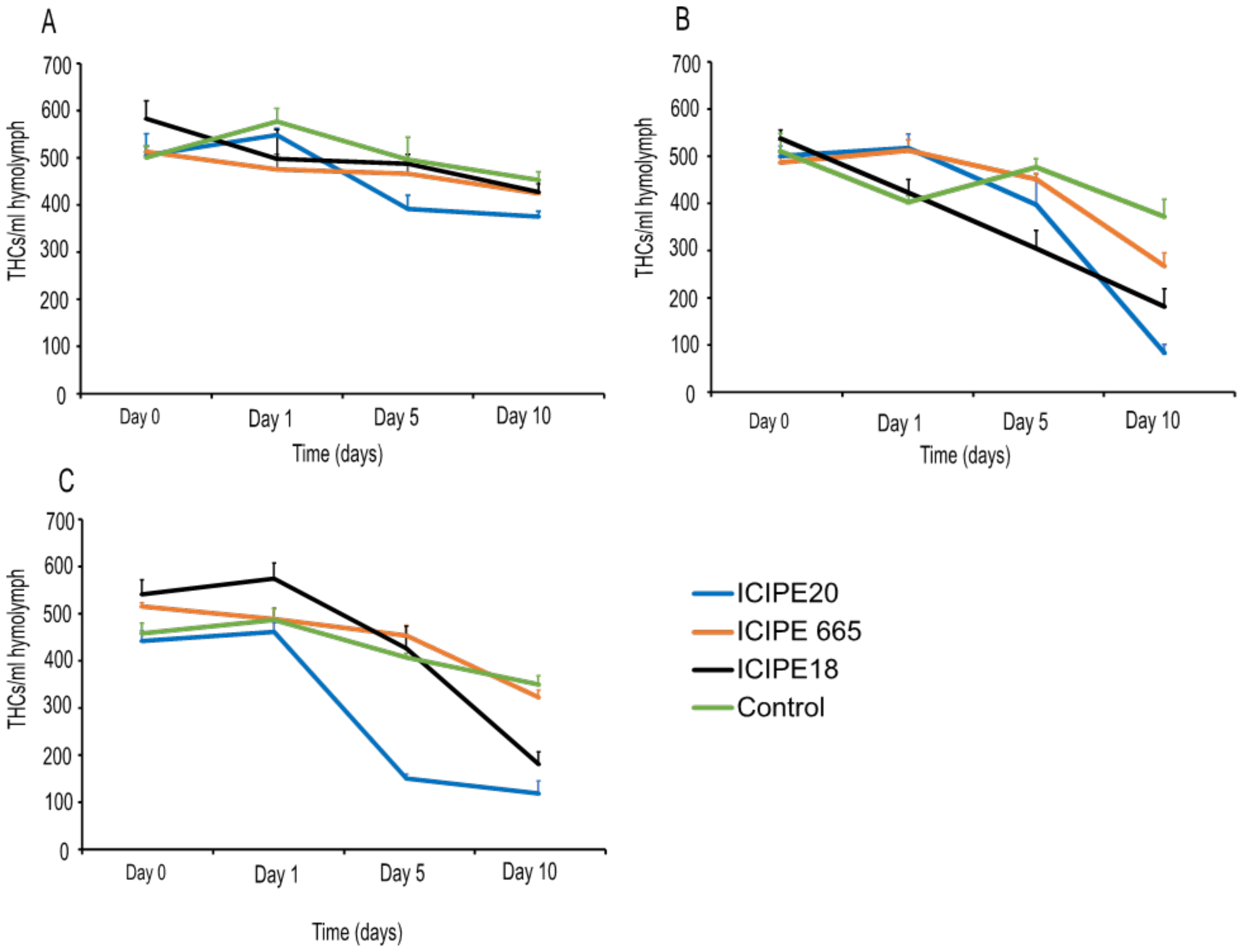

Supplementary Figure 4: Effect of *Metarhizium anisopliae* isolates and temperature on THC of *Phthorimaea absoluta* adults.

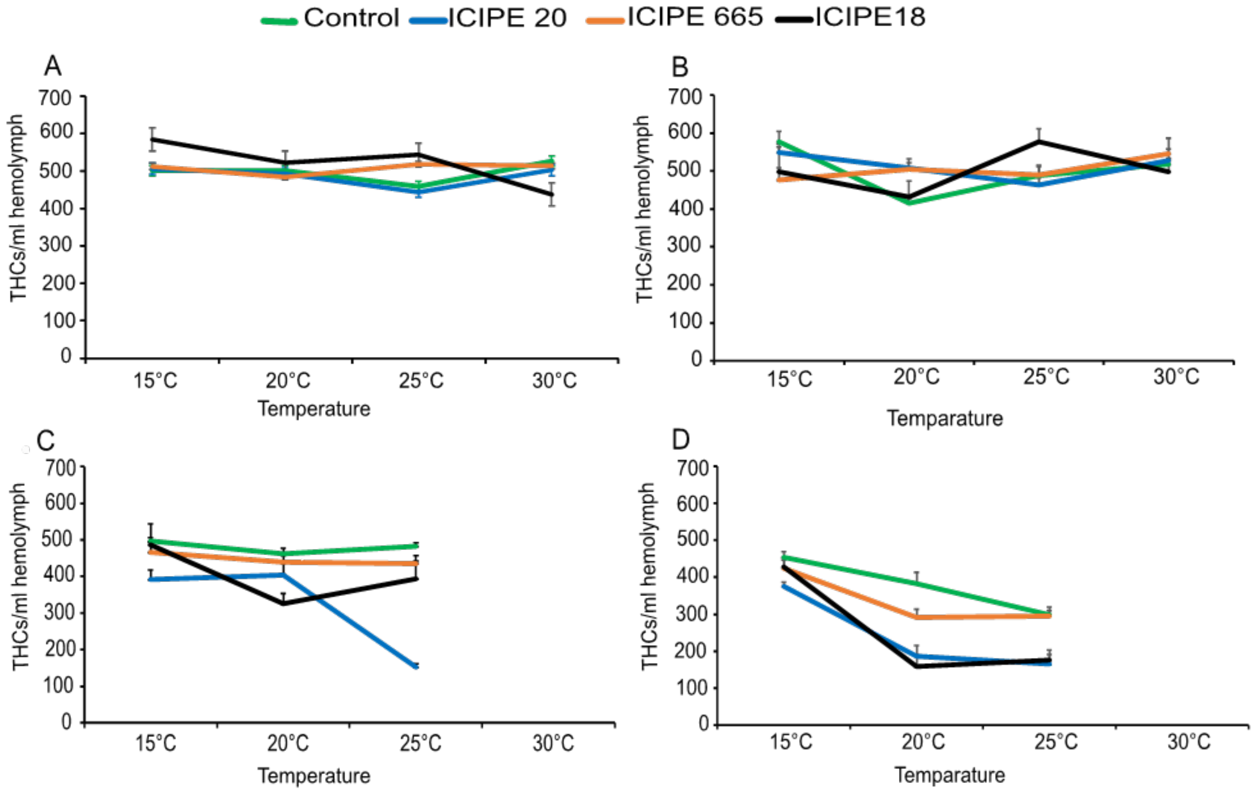

Supplementary Figure 6: Effect of *Metarhizium. anisopliae* isolates and temperature on THC of *Phthorimaea absoluta* adults.

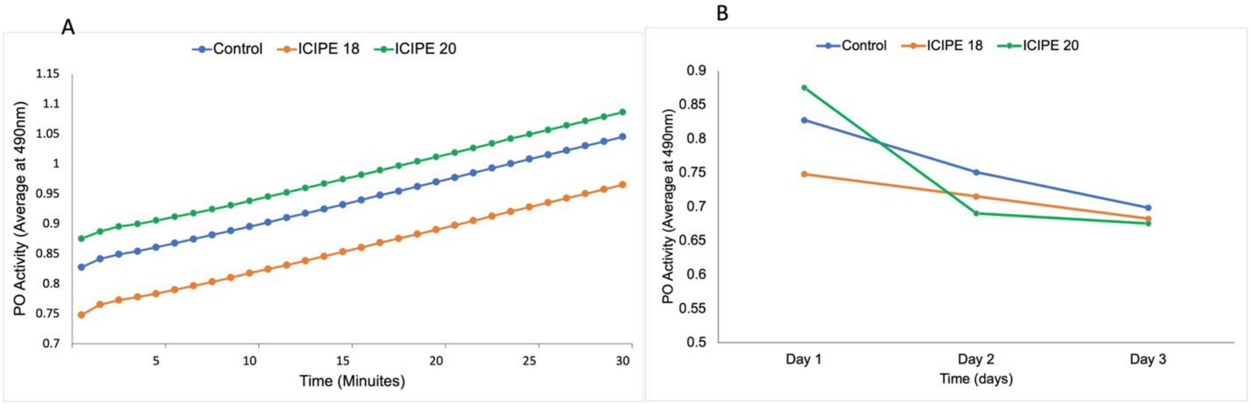

Supplementary Figure 6: Phenoloxidase activity, optical density (OD) average at 490nm in *Phthorimaea absoluta* hemolymph treated with *Metarhizium anisopliae* isolates, over time (A) Evaluated at 24 hours after infection (B) Optical density trend over three days.

frontiers

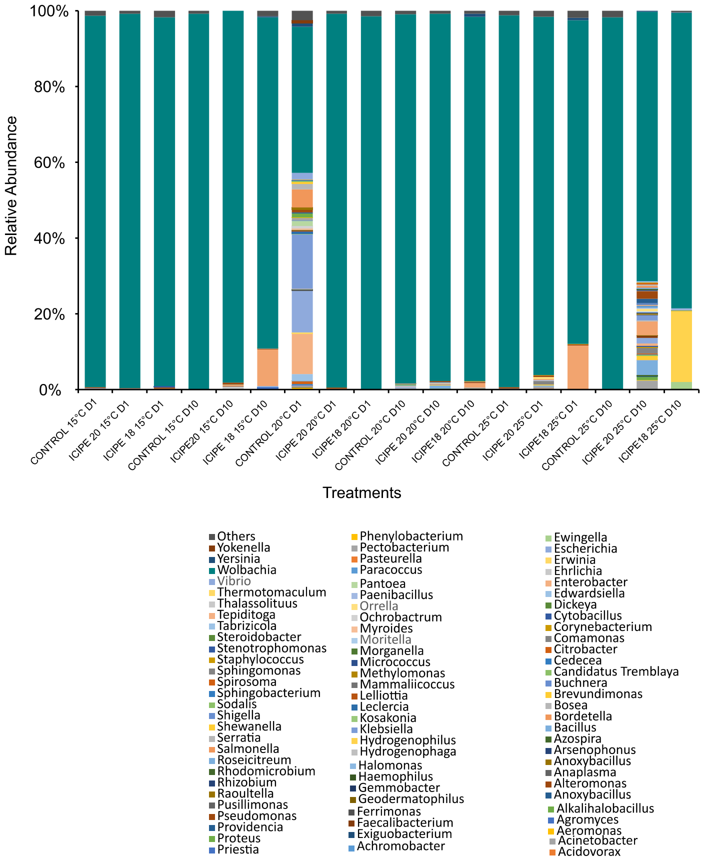

Supplementary Figure 7: Effect of *Metarhizium anisopliae* isolates (ICIPE 18 and ICIPE 20) on *Phthorimaea absoluta* gut microbiota at different temperature regimes
